# NETosis‐Like Response Triggered by Extracellular Vesicle (EV)‐Delivered Viral Nucleic Acid, a Novel Cellular Immune Mechanism in Crustacean

**DOI:** 10.1002/jev2.70210

**Published:** 2025-12-28

**Authors:** Hang Hu, Chunyi Zhong, Xinshan Zhao, Cheng Yi, Yi Gong

**Affiliations:** ^1^ School of Life Sciences Nanchang University Nanchang China; ^2^ Key Laboratory for Aquatic Germplasm Innovation and Utilization of Jiangxi Province Nanchang University Nanchang China

**Keywords:** extracellular vesicle, innate immunity, NETosis‐like response, P38‐MAPK signal pathway, viral infection, wsv271

## Abstract

As a novel identified manner of cell death, NETosis is widely regarded as an effective approach to resist pathogen infection but mainly focused on vertebrates with systematic cell typing. Besides, the role of extracellular vesicles (EVs), which are essential tools for intercellular information exchange, in regulating NETosis during pathogen infection has yet to be addressed. Here, we found that viral mRNA wsv271 could be packaged by EVs secreted by haemocytes during WSSV infection in mud crab, and delivered to the neutrophil‐like cells, followed by translation into viral protein, and then interacted with the TIR domain of Toll4 to recruit MyD88, so as to activate P38‐MAPK signal pathway and further facilitate PAD4 phosphorylation and nuclear translocation to mediate histone‐H3 citrullination, which eventually activated NETosis‐like response in haemocytes to suppress the spread of viral infection. Therefore, our research not only identified neutrophil‐like cells from the haemocytes of a crustacean based on single‐cell transcriptomics but also revealed a novel NETosis induction mechanism mediated by EVs‐derived viral nucleic acid delivery.

## 1 Introduction

Extracellular vesicles (EVs) refer to the membrane‐enclosed structures that can be released by almost all cell types (Kalluri and LeBleu 2020). These nanosized vesicles are mainly derived from multivesicular bodies (MVBs) formed by the inward budding of intracellular lysosomes (Trajkovic et al. 2008). Through packaging various bioactive molecules, including proteins, lipids and nucleic acids, EVs can function as the crucial mediator for intercellular communication by inducing phenotypic changes in recipient cells (Ostrowski et al. 2010). From current knowledge, there is evidence that EVs participate in the regulation of a variety of physiological and pathological processes, such as the immune responses, tissue repair and cardiovascular diseases (Van Niel et al. 2022). For example, it is reported that EVs secreted from antigen‐presenting cells (APCs), such as dendritic cells (Li et al. 2022), macrophages (Li et al. 2021) and B cells (Yang et al. 2021), could transmit loaded antigens onto major histocompatibility complex (MHC) to activate T cells and initiate immune responses, which played essential roles in coordinating and amplifying immune responses by facilitating the transport of immune‐related molecules between cells (Tassetto et al. 2017). Considering that the biogenesis and function of EVs are highly conserved in animals (Ofir‐Birin and Regev‐Rudzki 2019; Nikonorova et al. 2022), it is feasible to investigate the evolution of biological functions by comparing the molecular composition, functions and mechanisms of EVs among different organisms.

Unlike other forms of cell death, such as apoptosis and necrosis, NETosis, also known as programmed cell death mediated by NETs (neutrophil extracellular traps) (Yipp et al. 2012), is a type of cellular immune response activated by releasing a mesh structure composed of chromatin DNA fibres, histones and antimicrobial proteins outside the cell to trap and eliminate pathogens (Brinkmann et al. 2004; Branzk et al. 2014). NETosis inducers such as bacteria, fungi and viruses can promote the transcription and translation of myeloperoxidase (MPO), neutrophil elastase (NE) and peptidyl arginine deaminase 4 (PAD4); then PAD4 translocate to the nucleus to citrullinate histones and subsequently leads to alterations in chromatin structure and the unwinding of chromatin DNA (Papayannopoulos). After that, with the degradation of nuclear membrane, granules composed of NE1, MPO and other cytoplasmic antibacterial proteins associate with depolymerized chromatin DNA to form a microbicidal mesh structure and are released into the extracellular space (Urban et al. 2009; Metzler et al. 2014). These substances and histones bind with chromatin DNA and are often used as indicators of NETosis. In addition, reactive oxygen species (ROS) accumulation induced by oxidative burst in activated neutrophils is also regarded as a crucial indicator for NETs formation (Kenny et al. 2017). It has been reported that extracellular trapping net structures were observed within haemocytes in shore crab challenged with bacterial infection, cell death induced under these conditions is referred to as ETosis (Robb et al. 2014). Moreover, researchers found that the distribution of extracellular vesicles possesses a significant correlation with the citrullination levels of histones in shore crab during pathogen infection; the citrullination of histones also serves as the NETosis indicator in vertebrates (Coates et al. 2023). However, given that the haemocytes of crustaceans are composed of numerous cell types with distinct immune functions (Söderhäll et al. 2022), there is no definitive conclusion regarding specific cell types that undergo ETosis; the molecular mechanisms underlying its activation also remain unexplored.

The open circulatory system is a typical characteristic of crustaceans, where haemocytes, nutrients and oxygen circulate together in the haemolymph (Wang and Wang 2015). In this context, EVs can be transported to nearly all tissues and organs to exert biological functions via this type of circulatory system (Söderhäll 2016). In addition, crustaceans belong to invertebrates, which resist pathogenic microorganism infection and mainly rely on the innate immune system (Huang et al. 2020); thus, it is feasible to eliminate the influence of acquired immunity on the infection process (Chen and Wang 2019). These considerations indicate that crustaceans are more suitable biological systems for EVs to mediate innate immune responses compared to vertebrates. However, as a topical research area, research about how EVs participate in the antiviral immune response is predominantly focused on higher organisms (Kalluri 2024); there is a strong possibility to obtain novel immunological knowledge through the study of EVs‐mediated innate immunity in invertebrate research models.

In an effort to systematically elucidate the role of EVs‐dependent regulatory pathway in modulating host innate immune response during viral infection, we employed mud crab *Scylla paramamosain* infected with white spot syndrome virus (WSSV) as the research model (Li and Wang 2021). WSSV is highly pathogenic to crustacean, white spot syndrome caused by WSSV is the Class II animal diseases in China, so far, there is no specific and effective intervention to mitigate with WSSV‐related diseases (Sun et al. 2023). Our findings indicated that viral nucleic acid *wsv271* packaged within EVs could be delivered to the neighbouring neutrophil‐like cells and further activate NETosis‐like response to suppress viral infection. Therefore, our findings reveal a novel EVs‐dependent NETosis induction and regulation mechanism, which not only expands our understandings of innate immune response mediated by EVs but also provides cellular targets to cope with viral infection in the aquaculture industry.

## 2 Materials and Methods

### 2.1 Ethics Statement

The mud crabs used were taken from Niutianyang aquaculture farm (Shantou, China). No specific permits were required since the mud crab did not belong to an endangered or protected species. The animals were processed according to ‘the Regulations for the Administration of Affairs Concerning Experimental Animals’ established by the Jiangxi Provincial Department of Science and Technology on the Use and Care of Animals.

### 2.2 WSSV Challenge and Detection

Healthy mud crabs, about 50 g each, were domesticated for 72 h in a constant temperature culture tank at 25°C and 20‰ salt seawater. First, PCR was performed with WSSV‐specific primers (5’‐TATTGTCTCTCCTGACGTAC‐3’ and 5’‐CACAT‐TCTTCACGAGTTAC‐3’) to ensure that the mud crabs used were WSSV‐free prior to treatment. Then, each mud crab was injected with 200 µL of WSSV suspension (1×10^6^ particles/mL), the viral dose was referred to published studies (Tran et al. 2022; Wu et al. 2007). After different periods of injection, the haemolymph and tissues of 9 randomly selected mud crabs were collected for later use. Premix Ex Taq (Probe qPCR) (Takara, Japan), WSSV‐specific primers (5’‐TTGGTTTCATGCCCGAGATT‐3’, 5’‐CCTTGGTCAG‐CCCCTTGA‐3’) and TaqMan probe (5’‐FAM‐TGCTGCCGTCTCCAA‐TAMRA‐3’) were used for RT‐qPCR to detect WSSV copies.

### 2.3 Isolation and Purification of EVs

In order to isolate EVs from the haemolymph of mud crabs, the haemolymph was centrifuged at 8000 × *g* for 30 min at 4°C, then the supernatant was centrifuged at 20,000 × *g* for 1 h at 4°C, after that, the above supernatant was centrifuged at 130,000 × *g* for 2 h at 4°C. Finally the collected sediment was suspended with 2 mL of 0.95 M sucrose solution, and 2 mL of 2, 1.3 and 0.95 M sucrose solution were successively added into the suspension, followed by centrifugtion at 200,000 × *g* for 16 h at 4°C. The solution between 1.3 M and 0.95 M sucrose was collected and filtered with a 0.22‐µm filter membrane and then dialyzed with an 8–15‐kDa dialysis bag for 3 h. The obtained EVs were stained with 2% phosphotungstic acid and observed by transmission electron microscopy (Leica EM UC7, Germany) and quantified through Nanoparticle Tracking Analysis (NTA) (Malvern Instruments, UK).

### 2.4 RNA Interference Assay

Based on the sequence of wsv001 (AY048547.1), wsv091 (JX515788.1), wsv271 (MH663976.1), wsv277 (MN840357.1), wsv447 (KT995472.1), GFP (LN515608.1), ERK1 (KC342028.1), ERK2 (MN412584.1), P38 (KC711047.1), Toll4 (JX680257.2), MyD88 (GQ847862.1) and PAD4 (A0AAW0UK75), the siRNAs targeting above genes were designed by BLOCK‐iT RNAi Designer (rnaidesigner.thermofisher. com) (Table S1). In vitro Transcription T7 Kit (Takara, Japan) was used to synthesize specific siRNAs targeting the above genes according to the user's instructions. Then, 1 µg of siRNAs was injected per gram of mud crab. After different periods of injection, 3 mud crabs for each group were randomly selected and stored for later use. The crabs injected with GFP‐siRNA were set as the control group.

### 2.5 Quantification of mRNA With RT‐qPCR

Total RNA was extracted from haemocytes (*n* = 6 biological replicates; 6 individual crabs) using RNAiso Plus (Takara Bio, Shiga, Japan). First‐strand cDNA was synthesized from 1 µg total RNA with PrimeScript RT reagent kit (Takara Bio) using oligo‐dT primers. Quantitative PCR was performed in triplicate (Technical replicates) with TB Green Premix Ex Taq II (Takara Bio) on a QuantStudio 5 system (Applied Biosystems). Each 20 µL reaction contained 10 µL TB Green mix, 0.8 µL each primer (10 µM), 2 µL cDNA template(5× diluted)and 6.4 µL nuclease‐free water. The number of PCR cycles was set as 35. All oligos were synthesized by TsingKe Biotech (Beijing, China) with HPLC purification. Relative fold change of mRNA levels was determined by using the 2^−ΔΔCt^ algorithm. Statistical significance was determined by ANOVA across biological replicates. β‐actin served as the reference gene.

### 2.6 Western Blot Analysis

The samples were lysed by using RIPA buffer containing 1 mM PMSF, and denatured for 5 min at 95°C, followed by centrifugation at 13,000 × *g* for 5 min at 4°C. Then the supernatant was mixed with 5 × SDS loading buffer and separated by 12% SDS‐polyacrylamide gel and transferred onto a nitrocellulose membrane (Millipore, USA). Then, the membranes were blocked with Quick‐Block Western (Beyotime, China) and incubated with primary antibodies overnight at 4°C. After washing with TBST, the membrane was incubated with secondary antibody (anti‐rabbit IgG [SE134, Solarbio], anti‐mouse IgG [SE131, Solarbio]) for subsequent detection by ECL substrate (Thermo Scientific, USA). Primary antibodies used were listed as follows: CD63 (Catalogue no: K007602P), CD9 (K002936P, Solarbio), TSG101 (K006850P, Solarbio), Calnexin (K000499P, Solarbio), Tubulin (K200059M, Solarbio), MPO (AG2657, Beyotime), PTX3 (A12669, ABclonal), histone‐H2A (AF1711, Beyotime), histone‐H3 (AF0009, Beyotime), MyD88 (AF2116, Beyotime), ERK1 (AF1315, Beyotime), ERK2 (AF1255, Beyotime), Toll4 (A5258, Abclone), NE1 (A8953, Abclone), PAD4 (A25102, Abclone), Tyr, Ser and Thr phosphorylation (ICP9805, ICP9806, ICP9807, Immunechem), histone‐H3 (citrulline R2+R8+R17) (ab281584, Abcam). P38 and wsv271 antibodies were prepared in our laboratory. When conducting Western blot detection of total cellular protein, Tubulin is used as the internal control protein. During the Western blot detection of cytoplasmic proteins, Tubulin is used as the internal control protein. During the Western blotting of cells and proteins, histone H3 is used as the reference protein. When detecting EV proteins, CD63 is used as the reference protein.

### 2.7 MPO‐DNA and Histone‐H3 ELISA Assay

Haemocytes were collected and lysed with RIPA lysis buffer (Beyotime, China) (200 µL/10^6^ cells), followed by protein concentration measurement through BCA method. The MPO‐DNA ELISA kit (JingMei, China) and citrullinated histone‐H3 ELISA kit (Ivdshow, China) were used according to the instructions. 100 µL of protein lysate and corresponding substrate were crosslinked and incubated in 96‐well plate at room temperature for 30 min, after washing with cleaning buffer, 100 µL of immunoassay compound solution was added and incubated for 10 min, subsequently, 50 µL of colour‐substrate solution was added, and the light absorption at 450 nm was detected by microplate reader (SpectraMax i3, USA).

### 2.8 Confocal Microscopy Imaging of HE‐Stained Gill Sections

The gill filaments of crabs injected with PBS, WSSV, EV‐PBS and EV‐WSSV were dissected and immersed in 4% paraformaldehyde for fixation. Following this step, the specimens were held at 4°C for a period of 24 h to preserve the intricate layered structure. The dehydration process was conducted using a graded ethanol series (50% to 100%; each concentration maintained for 2 h), after which drying was conducted with a transition from xylene‐ethanol (50% xylene/ethanol to 100% xylene; repeated three times for 30 min each). The paraffin embedding was performed using low‐melting‐point wax (Paraplast X‐tra, McCormick) at 52°C under 500 mmHg vacuum conditions for 2 h. The sections (5 µm thickness) were cut perpendicular to the axis of the gill arches, placed on positively charged slides, and dried at 40°C for 12 h. After HE staining, the sections were made to have refractive index matching by immersing in an anti‐fading mounting medium.

### 2.9 Fluorescence In Situ Hybridization

Mud crab haemocytes were plated onto confocal culture dishes with 10% polyacid‐resistant pretreatment and then fixed with 4% paraformaldehyde for 15 min at room temperature. After being dehydrated with 70% ethanol and incubated overnight at 4°C, hybrid solution [1 × SSC (15 mM sodium citrate, 150 mM sodium chloride, pH 7.5 and 10% (w/v) dextran sulphate] was added and incubated for 30 min, followed by incubation with 500 mM fluorescent probe of wsv271 (Table S1) for 16 h at 4°C. After being washed with PBS 3 times and stained with DAPI dye solution (4’,6‐diamidino‐2‐phenylindole) (50 ng/mL) (Sigma, USA) for 5 min, the culture dishes were observed with confocal microscope (TCS SP803040101, Leica, Germany).

### 2.10 Sample Preparation for Scanning Electron Microscopy

The haemocytes were fixed in 0.1 M PBS (pH 7.4) containing 2.5% glycerol and 2% formaldehyde at 4°C for at least 2 h. Subsequently, the samples were washed three times with PBS and dehydrated successively through varying concentrations of ethanol (30%, 50%, 70%, 90% and 100% ethanol; each step for 15 min) and subjected to two 20‐min treatments with anhydrous ethanol. The specimens were then dried utilizing supercritical carbon dioxide (Leica EM CPD300) at 31.1°C and 72.9 pascals. The dried samples were fixed on aluminium slides with carbon tape and coated with 10 nm gold‐palladium (Quorum Q150T ES). Images were taken using a secondary electron detector (Zeiss GeminiSEM) with an accelerating voltage of 10 kilovolts and a working distance of 8–12 mm. Uninfected cells served as the control group.

### 2.11 Flow Cytometry Cell Sorting Based on PTX3 Labelling

Adjust the haemocytes to a concentration of 1×10⁷ cells/ml and resuspend them in PBS solution, and then incubate the cells with the Alexa Fluor 488‐conjugated non‐specific anti‐PTX3 antibody (diluted 1:50) at 4°C for 30 min in the dark. After being washed three times with cold sorting buffer (PBS + 0.5% BSA + 2 mM EDTA), the sample is filtered through a 35‐micron nylon mesh and kept at 4°C before sorting. Sorting is performed using the BD FACSAria III sorter, with an excitation wavelength of 488 nm, a nozzle diameter of 90 µm and a pressure of 45 pounds per square inch. PTX3⁺ cells are collected based on an emission intensity readout from Alexa Fluor 488 emission intensity that is higher than 10⁴ (triggered by the FSC‐H/SSC gate of live lymphocytes, purity mode >98%). Post‐sorting validation involves reanalysis of the sorted fraction to ensure that the purity level of target cells surpasses 95%, with three biological replicates performed for each condition.

### 2.12 Virus‐Carrying Cells Detection

WSSV particles were purified with a universal virus concentration kit, then the isolated viral particles were stained with SYBR green fluorescent dye for 2 h at 4°C, and the final concentration was adjusted to 10^7^ particles/µL to ensure an adequate quantity of viral particles for capture by haemocytes. After that, 100 µL of SYBR green‐stained WSSV particles were injected into mud crabs, and haemocytes were collected in the dark at 3 h post‐injection. Subsequently, the haemocytes were plated onto confocal culture dishes and observed with confocal microscope (TCS SP803040101, Leica, Germany). In addition, the haemocytes were also subjected to flow cytometry detection (BD Accuri C6 Plus, USA), and the data were analyzed by FlowJo 10.

### 2.13 Single‐Cell RNA Sequencing

For library preparation, gel beads‐in‐emulsion (GEMs) embedded with UMIs and cell barcodes were used to isolate single cells. Post‐lysis, polyA RNA was hybridized to beads for reverse transcription, generating cDNA tagged with UMI and cell barcode at the 5’end. After that, libraries were constructed via second‐strand cDNA synthesis, adaptor ligation and universal amplification, enriching 3’ transcripts with cell barcode and UMI. Followed by quantification using high sensitivity DNA chip and qubit assay and sequenced on DNBSEQ‐T7 (PE150, v3.0 kit). The raw data have been uploaded to NCBI public database (accession number: GSE289112). For data processing, reads were processed by Cell Ranger (v7.1.0) and aligned to GRCm38 genome via STAR. Gene‐barcode matrices were generated by UMI counting and non‐cell barcode filtering. Low‐quality cells were removed using Seurat (v4.1.1) (criteria: transcript/gene counts, mitochondrial gene percentage). Then, data normalization, variable gene identification and multi‐sample integration were performed via dataset ‘anchors’. PCA reduced dimensions to top 30 components, followed by t‐SNE clustering visualization.

### 2.14 Identification of Cell Types and Subtypes by T‐SNE

Cells were clustered using graph‐based clustering of the PCA‐reduced data with the Louvain Method after computing a shared nearest neighbour graph. For sub‐clustering, we applied the same procedure of scaled dimensionality reduction and clustering to the specific set of data (usually restricted to one type of cell). For each cluster, we used the Wilcoxon Rank‐Sum Test to find significantly differentially expressed genes comparing the remaining clusters. SingleR and known marker genes were used to identify cell type.

### 2.15 Transcriptome Sequencing

The transcriptome of EVs isolated from mud crab was sequenced by Beijing Biomarker Biotechnology Co., LTD (China); the processing groups include PBS group and WSSV group, and the original data have been uploaded to NCBI public database (Accession number: PRJNA837743). The transcriptome of haemocytes collected from mud crabs treated with EVs‐PBS, EVs‐WSSV+GFP‐siRNA and EVs‐WSSV+wsv271‐siRNA was sequenced by Kidio Biotechnology Co., Ltd (Guangzhou, China); the raw data have been uploaded to NCBI public database (accession number: PRJNA1093225).

### 2.16 Statistical Analysis

Statistical analysis in this study was performed using Origin Pro 8.0 software: one‐way ANOVA for multi‐group comparisons (≥3 groups), *T*‐tests for two‐group comparisons and one‐sample *t*‐tests for reference value analyses; *p* value < 0.05 indicated statistical significance across all analyses, and all experiments included at least three biological replicates.

## 3 Results

### 3.1 EVs Suppress Viral Replication by Inducing NETosis‐Like Response During WSSV Infection

**FIGURE 1:**
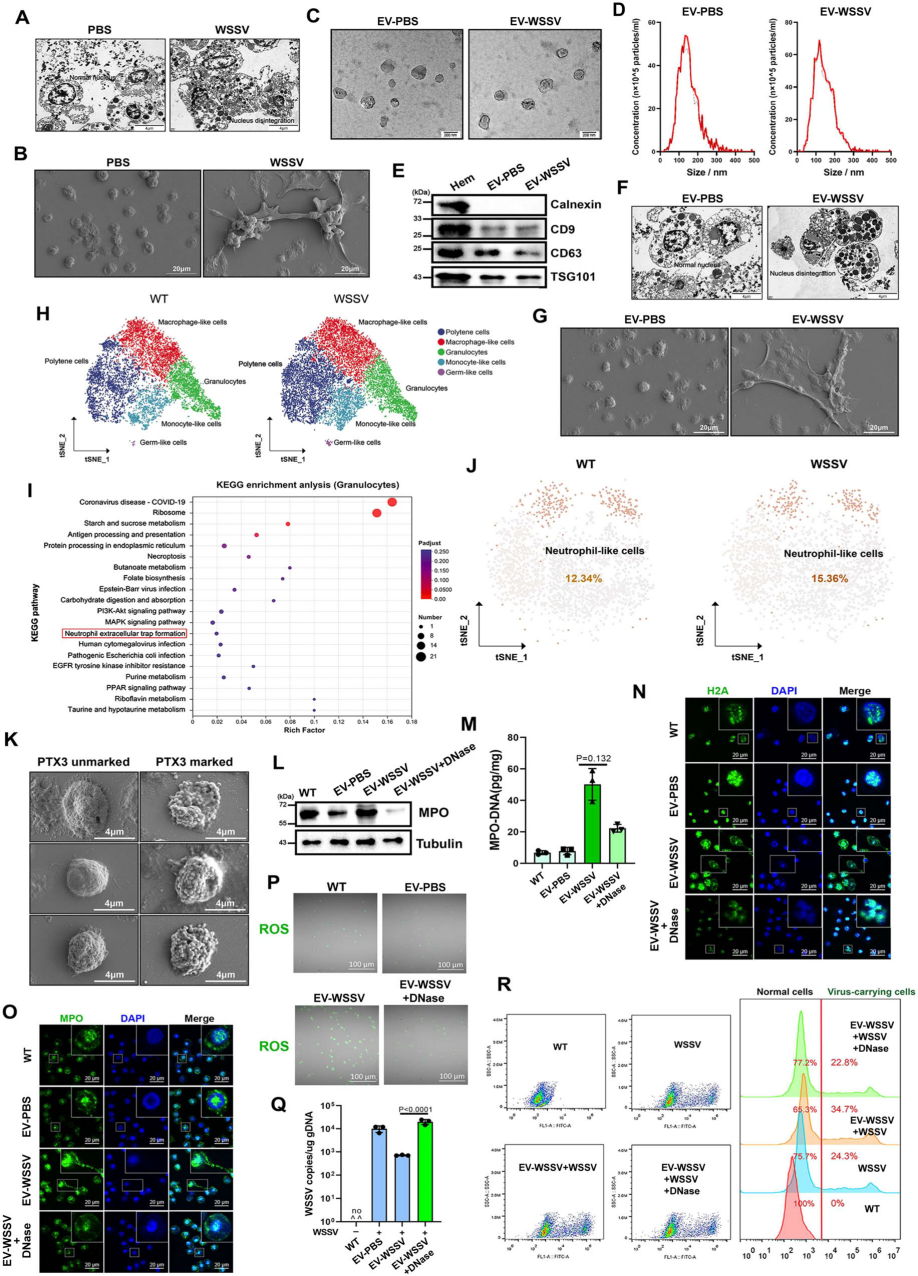
EVs suppress viral replication by inducing NETosis‐like response during WSSV infection. (A) The morphology of haemocytes in PBS or WSSV‐challenged mud crabs was detected by Transmission electron microscopy, scale bar, 4 µm. (B) The morphology of haemocytes in PBS or WSSV‐challenged mud crabs was detected by Scanning electron microscope, the trapping net in the WSSV injection group was clearly visible, while that in the PBS injection group was not. scale bar, 20 µm. (C‐D) Cup‐shaped EVs with a diameter of approximately 100 to 200 nm isolated from PBS or WSSV‐challenged mud crabs were detected by TEM (C) and NTA (D). (E) Western blot analysis of positive marker proteins (CD9, CD63 and TSG101) and cytoplasmic marker protein Calnexin as negative control in cell lysates and EVs. (F) The morphology of haemocytes in EVs‐PBS and EVs‐WSSV treated mud crabs, scale bar, 4 µm. (G) The morphology of haemocytes in EV‐PBS or EV‐WSSV‐injected mud crabs was detected by Scanning electron microscope, the trapping net in the EV‐WSSV injection group was clearly visible, while that in the EV‐PBS injection group was not. scale bar, 20 µm. (H) A t‐SNE plot showing five major cell types identified in scRNA‐ seq dataset (n = 21631 in total; WT, 9695; WSSV‐infected, 11936). (I) KEGG‐annotated bubble map of differentially expressed genes in granulocytes between WT and WSSV‐treated groups. The functions related to the formation of the neutrophil trapping net are marked with red boxes. (J) A t‐SNE plot showing the distribution and proportion of neutrophil‐like cell in granulocytes, WT 12.34%, WSSV 15.36%. (K) The morphology of the cells labelled with PTX3 antibody and the un‐labelled cells was observed using a scanning electron microscope. The positive cells showed obvious granular substances on their surface, while the negative cells did not, scale bar, 4 µm. (L‐M)The levels of MPO protein and MPO‐DNA complex in haemocytes after treated with EVs and DNase (NETosis inhibitor). MPO protein was detected by Western blot (L), tubulin was used as the internal control, MPO‐DNA complex were quantified by ELISA. The Ev‐W + DNase group serves as the negative control group. (M). (N‐O) The cellular localization of histone‐H2A (N) and MPO (N) proteins in haemocytes after treated with same treatment, DAPI was used to stain the nucleus, scale bar, 20 µm. In the cells of the EV‐WSSV treatment group, the H2A protein was located in the cytoplasm, and MPO was located on the trapping net. (P) The ROS levels in haemocytes after treated with EVs and DNase were detected by fluorescence microscope, DCFH‐DA was used as fluorescence probe and the scale bar was 100 µm. The fluorescence of the cells in the EV‐WSSV treatment group was the highest among all the control groups. (Q) Virus copy numbers in WSSV‐challenged mud crab after treated with EVs and DNase were detected by probe‐qPCR. The WT group served as the negative control, while EV‐PBS was used as the positive control. The viral copy number in the EV‐WSSV group was approximately 10^3^ copies/ng DNA. (R) The proportion of virus‐carrying haemocytes in WSSV‐challenged mud crabs after same treatment were detected by flow cytometry, SYBR Green was used to stain viral particles. All data represented were the mean ± SD of at least three independent experiments.

To explore the antiviral immune mechanism in crustaceans, a WSSV‐infected mud crab model was constructed. It was observed that the nucleus of haemocytes was disintegrated in mud crabs during WSSV infection (Figure 1A), which belongs to the typical characteristic of NETosis in vertebrates (Yipp et al. 2012; De Bont et al. 2018). Besides, we also observed a typical morphology of the extracellular trapping net in the haemocytes under an SEM (scanning electron microscope) (Figure 1B). Considering the crucial role of EVs in innate immune regulation, we isolated EVs from mud crabs before/after WSSV infection, the cup‐shaped structure of EVs collected from PBS‐injected (EV‐PBS) and WSSV‐injected (EV‐WSSV) mud crabs were observed under TEM (transmission electron microscopy) (Figure 1C), the sizes and concentration of the EVs were measured by NTA (nanoparticle tracking analysis) (Figure 1D), and the purity were ascertained by EVs markers CD9, CD63, TSG101 and ER membrane marker Calnexin (Figure 1E). We found that EV‐WSSV injection alone could also lead to nucleus disintegration and extracellular trapping net in WSSV‐free mud crabs (Figure 1F,G). The HE staining of the gill filaments also revealed that WSSV and EV‐WSSV could induce the formation of an extracellular trapping net (Figure S1A). Then, scRNA‐seq was conducted to examine the existence of neutrophils in mud crabs. A total of five major cell types were identified within haemocytes (Figure 1H), including polytene cells (33.6%), macrophage‐like cells (30.06%), granulocytes (24.20%), monocyte‐like cells (11.84%) and germ‐like cells (0.30%). These cell types were annotated based on the potential functions suggested by specific marker genes (Figure S1B, Supporting Information 1 and 2). It is worth noting that GO and KEGG analyses showed that granulocytes possess neutrophil extracellular trap formation function (Figure 1I, Supporting Information 3 and 4). Therefore, we further conducted subcellular typing on granulocytes (Figure S1C,D), PTX3 and crustin were used as marker genes of neutrophil‐like cells based on published studies (Jaillon et al. 2007; Segal 2005; Hernández‐Pérez et al. 2020). It was found that the proportion of neutrophil‐like cells was increased during WSSV infection (Figure 1J, Figure S1E,F, and Supporting Information 5 and 6). Then, the haemocytes were labelled with PTX3 antibody and detected via flow cytometry and confocal microscopy (Figure S1G,H). The results showed that PTX3‐marked haemocytes possess significant morphological differences compared with unmarked haemocytes under SEM (Figure 1K).

To confirm the above NETosis phenomenon, we detected a series of classical indicators after EV treatments (Figure S2A). The levels of MPO protein and MPO‐DNA complex were both significantly increased in EV‐WSSV group compared with EV‐PBS group (Figure S2B,C), while decreased when co‐injected with DNase (a widely used NETosis inhibitor) (Figure 1L,M, and Figure S2D). Besides, immuno‐fluorescence (IF) analysis showed that histone‐H2A, MPO and NE1 proteins were distributed diffusely in haemocytes after EV‐WSSV treatment (Figure 1N,O and Figure S2E,F). We defined the above phenomenon in mud crab as NETosis‐like response. Then, to explore the immunological significance of EVs‐mediated NETosis‐like response, ROS levels in haemocytes were measured by fluorescence microscope and microplate reader; the results showed that ROS levels were positively correlated with NETosis‐like response levels (Figure 1P and Figure S2G). Then, to evaluate the effect of EVs‐mediated NETosis‐like response on viral infection, the viral copy number was measured and found to be significantly reduced in haemocytes after EV‐WSSV treatment (Figure 1Q). Furthermore, through injection of SYBR green‐stained WSSV particles, we found that the proportion of virus‐carrying cells was notably increased (Figure 1R and Figure S2H), indicating that NETosis‐like response in haemocytes could enrich and eliminate viral particles. The survival rate analysis also indicates that EVs can reduce host death caused by viral infection (Figure S2I). Taken together, the above data suggest that EVs can induce NETosis‐like response in haemocytes, release ROS and inhibit virus replication during WSSV infection.

### 3.2 EVs Regulate NETosis‐Like Response and Viral Replication via Packaging wsv271 mRNA

**FIGURE 2:**
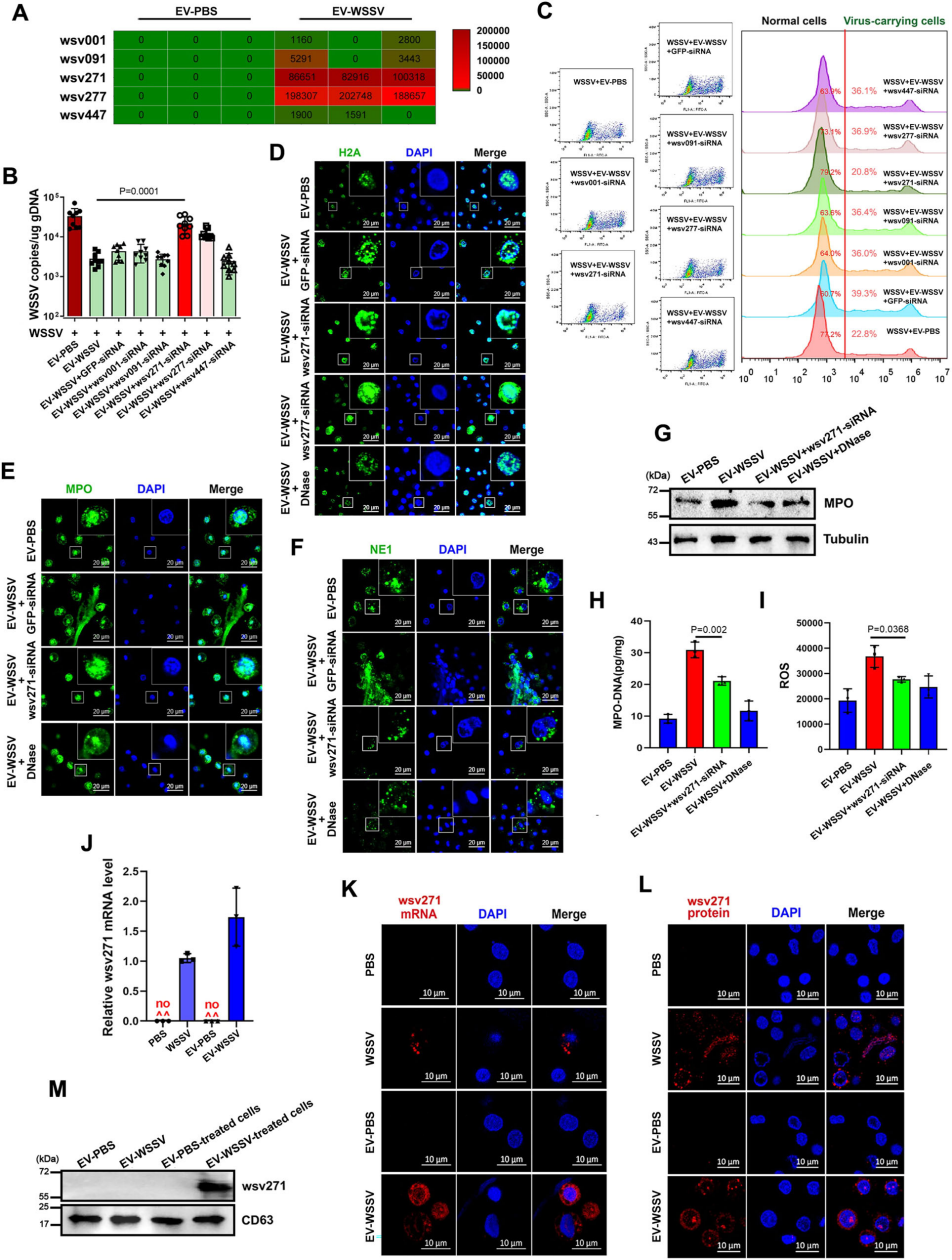
EVs regulate NETosis‐like response and viral replication via packaging wsv271 mRNA. (A) WSSV nucleic acids were identified from the transcriptome sequencing data of EVs via blasting with WSSV genome. The mRNA of wsv271 and wsv277 has a higher expression level of viral nucleic acids compared to other viruses. (B and C) The effects of EVs‐containing viral nucleic acids on EVs‐mediated virus suppression. EVs and siRNAs targeting viral nucleic acids were co‐injected into WSSV‐challenged mud crabs for 24 h, then viral copy numbers were detected via RT‐qPCR (B), and the proportion of virus‐carrying haemocytes was analyzed by flow cytometry (C). The WSSV + EV‐PBS group served as the negative control, while the WSSV + EV‐WSSV + GFP‐siRNA group served as the positive control. (D–F) The cellular localization of histone‐H2A (D), MPO (E) and NE1 (F) proteins in haemocytes after same treatment. DAPI was used to stain the nucleus, scale bar, 20 µm. In the cells of the EV‐WSSV + wsv271‐siRNA treatment group, the H2A protein was not located in the cytoplasm, and MPO, NE1 were located in the cytoplasm. (G and H) The levels of MPO protein and MPO‐DNA complex in haemocytes after same treatment. MPO protein was detected by Western blot (G), tubulin was used as the internal control, MPO‐DNA complex was quantified by ELISA (H). (I) The ROS levels in haemocytes after same treatment were detected by microplate reader. (J) RT‐qPCR was used to detect the expression of wsv271 after PBS, WSSV, EV‐PBS and EV‐WSSV treatments. The PBS injection group served as the negative control, while the WSSV injection group was the positive control group. (K) The localization of wsv271 mRNA in haemocytes was detected by using FAM‐labelled wsv271 mRNA probe, the nucleus was stained with DAPI, scale bar, 10 µm. The PBS injection group served as the negative control, while the WSSV injection group was the positive control group. (L) The localization of wsv271 protein was detected via IF using wsv271‐specific antibody, scale bar, 10 µm. The PBS injection group served as the negative control, while the WSSV injection group was the positive control group. (M) Western blot was performed to detect the expression of wsv271 protein in the EVs, and in the haemocytes of mud crabs after same treatment, respectively. CD63 was used as the internal reference. Only the haemocytes of the crabs injected with EV‐WSSV showed a positive wsv271 band. Data represent the mean ± SD of three or more independent experiments.

In order to clarify the mechanism of EV‐WSSV‐mediated NETosis‐like response and viral suppression, transcriptome sequencing was performed on the above isolated EVs. Five WSSV‐specific nucleic acids (wsv001, wsv091, wsv271, wsv277 and wsv447) were identified in EV‐WSSV via blasting with WSSV genome (Be detected in at least two samples) (Figure 2A). Then, to reveal the immunological role of these virus‐derived nucleic acids packaged by EV‐WSSV, EVs and specific viral siRNAs were co‐injected into WSSV‐treated mud crab (Figure S3A–E), the viral copy number was significantly reverted back to 10^4^ copies/ng gDNA when wsv271 and wsv277 were silenced (Figure 2B), indicating the essential roles of wsv271 and wsv277 in EVs‐mediated viral suppression. Besides, we found that the proportion of virus‐carrying cells was decreased to 20.8% only when wsv271 was silenced (Figure 2C). Furthermore, through detecting classical indicators for NETosis, including the cellular localization of histone‐H2A, MPO and NE1 proteins, the levels of MPO protein, MPO‐DNA complex and ROS (Figure 2D‐I). The results showed that wsv271 could induce the release of trapping net structures in haemocytes (Figure 2D‐F), and enhance the levels of MPO and ROS (Figure 2G‐I), which is required for triggering EVs‐mediated NETosis‐like response in mud crabs.

To explore the mechanisms of wsv271 involved in EVs‐mediated antiviral immune response, the key question is to ascertain whether wsv271 can be transmitted into haemocytes along with EVs. First, RT‐qPCR was conducted to confirm the existence of wsv271 nucleic acid in EV‐WSSV (Figure 2J). Then, we injected EVs into virus‐free mud crab and then detected the existence of wsv271 mRNA and protein in haemocytes by fluorescence in situ hybridization (FISH) and IF. The results of FISH showed that wsv271 mRNA packaged by EVs can be transported into haemocytes (Figure 2K). Furthermore, it was found that wsv271 protein could also be detected by Western blot and IF analysis by injecting EV‐WSSV alone instead of virus particles (Figure 2L and Figure S3F,G), while EVs isolated from WSSV‐infected mud crabs did not carry wsv271 protein (Figure 2M), indicating wsv271 mRNA packaged by EVs could be translated into viral protein. Taken together, these results suggest that wsv271 mRNA packaged by EVs could be transmitted and translated into viral protein in haemocytes, which is required for EVs‐mediated antiviral immune response.

### 3.3 wsv271 Packaged by EVs Activates P38‐MAPK Pathway to Regulate NETosis‐Like Response and Viral Replication

**FIGURE 3:**
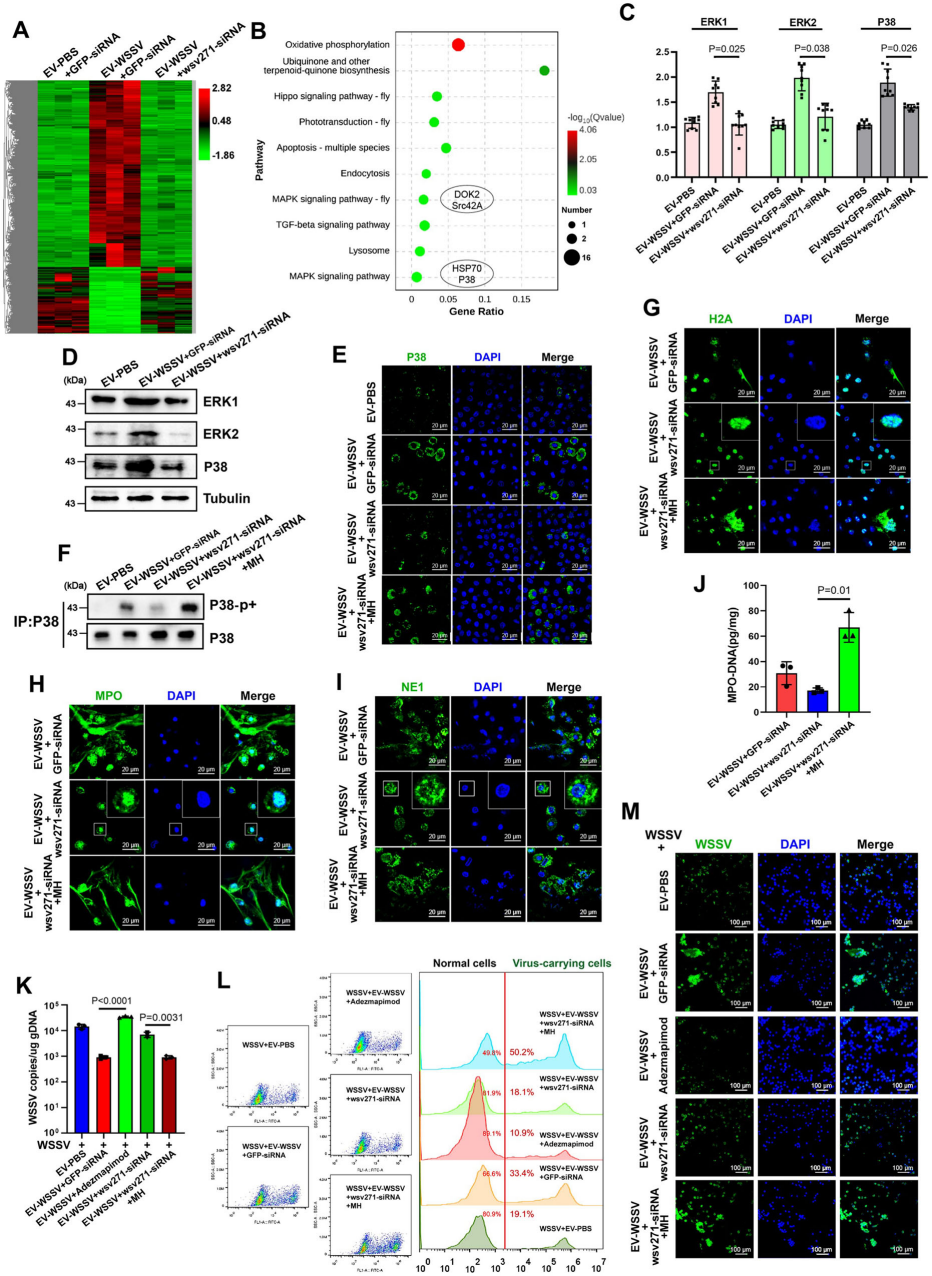
wsv271 packaged by EVs activates P38‐MAPK pathway to regulate NETosis‐like response and viral replication. (A) High‐throughput transcriptome sequencing of haemocytes after treated with EVs and wsv271‐siRNA, genes altered in EV‐WSSV injected group compared with EV‐PBS injected group, and with no significant difference when wsv271 was silenced, were shown on the heat map. (B) KEGG annotation highlighted the top ten signalling pathways relevant to the differentially genes shown in Figure 3A. The gene DOK2 and SrcA are enriched in the MAPK signalling pathway‐fly, while HSP70 and P38 are enriched in the MAPK signalling pathway. (C and D) RT‐qPCR and Western blot were used to detect the marker genes of MAPK pathway after treated with EVs and wsv271‐siRNA, including ERK1, ERK2 and P38, tubulin was used as the internal control. The EV‐WSSV‐siRNA injection group was used as the control group (E) IF analysis was conducted to reveal the level and distribution of P38 protein after treated with EVs, wsv271‐siRNA and MH (Metformin HCl, P38‐MAPK pathway activator), scale bar, 20 µm. The number of P38 positive cells increased in the MH injection group. (F) Western blot assay was performed to detect the phosphorylation level of P38 protein after same treatment, immunoprecipitated P38 was served as the internal control. The p38 phosphorylation signal in the wsv271‐siRNA injection group decreased. (G‐I) The cellular localization of histone‐H2A (G), MPO (H) and NE1 (I) proteins in haemocytes after same treatment. DAPI was used to stain the nucleus, scale bar, 20 µm. In the cells of the EV‐WSSV + wsv271‐siRNA + MH treatment group, the H2A protein was located in the cytoplasm, and MPO, NE1 was located in the extracellular trapping net. (J) The levels of MPO‐DNA complex in haemocytes after same treatment were quantified by ELISA. EV‐WSSV + wsv271‐siRNA injected group used as control. (K) Virus copy numbers in WSSV‐challenged mud crab after treated with EVs, wsv271‐siRNA, adezmapimod and MH were detected by RT‐qPCR. The MAPK signalling pathway inhibitor injection treatment group had a higher viral copy number compared to the EV‐WSSV injection group, while the MAPK signalling pathway inhibitor injection group had a lower viral copy number compared to the EV‐WSSV + wsv271‐siRNA injection group. (L‐M) The proportion of virus‐carrying haemocytes in WSSV‐challenged mud crabs after same treatment were detected by flow cytometry (L) and observed by fluorescence microscope (M), SYBR Green was used to stain viral particles, scale bar, 100 µm. Experiments were conducted in triplicate, with results presented as mean ± standard deviation.

To explore the mechanism of wsv271‐mediated NETosis‐like response activation, high‐throughput sequencing was performed on the haemocytes treated with EVs and wsv271‐siRNA (Fig. S4A). Among the differentially expressed genes, 676 genes were altered in EV‐WSSV group compared with EV‐PBS group, while showing no significant difference when wsv271 was silenced (Figure 3A), indicating that wsv271 was relevant to the expression of these genes. Then, the top ten KEGG enrichment annotations were selected. It is worth noting that two MAPK‐relevant pathways appeared in the top ten KEGG‐enriched pathways (Figure 3B), which also served as an important signalling pathway related to NETosis in vertebrates (Wang et al. 2019). Moreover, the results of RT‐qPCR and Western blot conducted on crucial genes in MAPK pathway (including ERK1, ERK2 and P38) showed that the mRNA and protein levels of ERK1, ERK2 and P38 were significantly increased after EV‐WSSV treatment, while this phenomenon was completely weakened when wsv271 was silenced (Figure 3C,D), indicating that EVs could activate MAPK pathway via wsv271. To further confirm these results, MH (Metformin HCl, P38‐MAPK pathway activator) was used (Figure S4B), and P38 phosphorylation, the essential feature during MAPK pathway activation, was detected. The results showed that both expression and phosphorylation levels of P38 protein were increased after EV‐WSSV treatment, while repressed when treated with wsv271‐siRNA (Figure 3E,F). These data suggested that EV‐WSSV could activate P38‐MAPK pathway via wsv271.

Then, to reveal the correlation between EVs‐mediated NETosis‐like response and P38‐MAPK pathway activation, NETosis indicators were detected, including the cellular localization of histone‐H2A, MPO, NE1 proteins and levels of MPO‐DNA complex. We found that wsv271‐mediated P38‐MAPK pathway activation could induce NETosis‐like response in haemocytes (Figure 3G‐J). Moreover, to evaluate the effects of wsv271‐mediated P38‐MAPK pathway activation on antiviral immune response, Adezmapimod (P38‐MAPK pathway inhibitor) was used (Figure S4C), the results showed that wsv271 packaged by EVs could suppress WSSV replication by activating P38‐MAPK pathway (Figure 3K), besides, the proportion of virus‐carrying cells was notably decreased to 10.9% when P38‐MAPK pathway was suppressed (Figure 3L,M). Taken together, these data demonstrate that wsv271 derived from EVs can activate P38‐MAPK signalling pathway to regulate NETosis‐like response and viral replication.

### 3.4 wsv271 Activates P38‐MAPK Pathway by Interacting With Toll4

**FIGURE 4:**
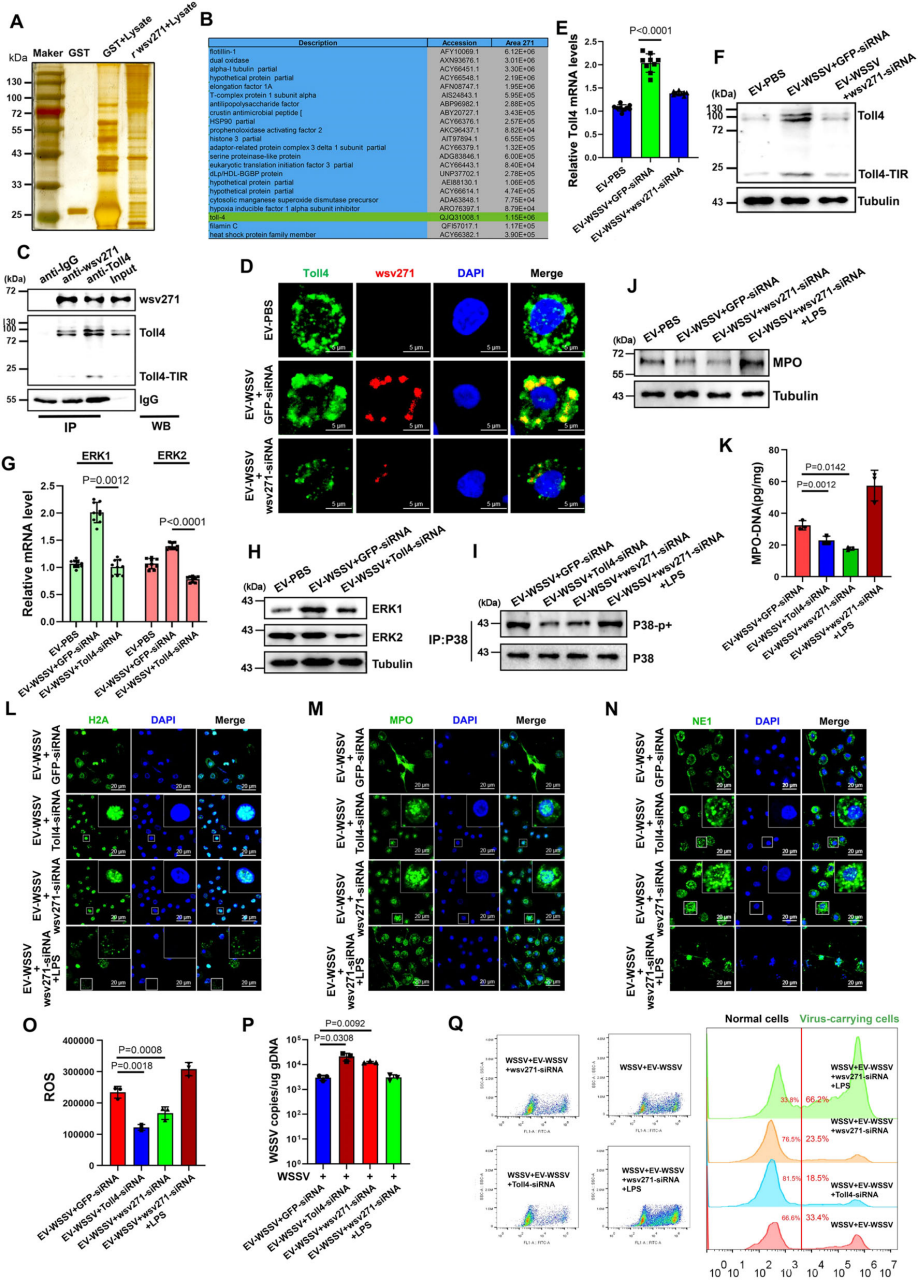
wsv271 activates P38‐MAPK pathway by interacting with Toll4. (A‐B) Identification of proteins bound to wsv271. The cell lysates of haemocytes were incubated with recombinant GST‐labelled wsv271 protein, and then treated with PreScission protease, followed by separated with SDS‐PAGE and silver staining (A), and identified by mass spectrometry (B). (C) The interactions between wsv271 and Toll4 in mud crab. Cell lysates were subjected to immunoprecipitation analysis with anti‐wsv271 IgG and anti‐Toll4 IgG, followed by Western blot using the indicated antibodies. The effluent from the anti‐IgG binding reaction was set as the negative control, while the cell lysate was used as the positive control. (D) Localization of wsv271 and Toll4 protein in mud crab haemocytes after treated with EVs and wsv271‐siRNA were detected by IF, DAPI was used to stain the nucleus, scale bar, 5 µm. The yellow fluorescence signal in Merge represents the co‐localization signal. The EV‐PBS injection group served as the negative control. (E‐F) RT‐qPCR and Western blot were performed to detect the mRNA and protein levels of Toll4 in haemocytes after treated with EVs and wsv271‐siRNA, tubulin was used as the internal control. The EV‐WSSV + wsv271 ‐ siRNA injection group was used as the control group. (G and H) RT‐qPCR and Western blot were performed to detect the mRNA and protein levels of ERK1 and ERK2 in haemocytes after treated with EVs and Toll4‐siRNA, tubulin was used as the internal control. The EV‐WSSV + GFP—siRNA injection group was used as the control group. (I) Western blot assay was performed to detect the phosphorylation level of P38 protein after treated with EVs, wsv271‐siRNA, Toll4‐siRNA and LPS (TLR4‐dependent P38‐MAPK pathway activator), immunoprecipitated P38 was served as the internal control. (J‐K) The levels of MPO protein and MPO‐DNA complex in haemocytes after treated with EVs, wsv271‐siRNA, Toll4‐siRNA and LPS. MPO protein was detected by Western blot (J), tubulin was used as the internal control, MPO‐DNA complex were quantified by ELISA (K). (L–N) The cellular localization of histone‐H2A (L), MPO (M) and NE1 (N) proteins in haemocytes after treated with EVs, wsv271‐siRNA, Toll4‐siRNA and LPS. DAPI was used to stain the nucleus, scale bar, 20 µm. (O) The ROS levels in haemocytes after treated with EVs, wsv271‐siRNA, Toll4‐siRNA and LPS were detected by microplate reader. (P) Virus copy numbers in WSSV‐challenged mud crab after treated with EVs, wsv271‐siRNA, Toll4‐siRNA and LPS were detected by RT‐qPCR. (Q) The proportion of virus‐carrying haemocytes in WSSV‐challenged mud crabs after treated with EVs, wsv271‐siRNA, Toll4‐siRNA and LPS were detected by flow cytometry, SYBR Green was used to stain viral particles. Results are presented as mean ± SD from three experiments.

In order to clarify how wsv271 regulates P38‐MAPK pathway, GST‐pull down was performed to screen the interacting proteins of viral protein wsv271 (Figure 4A). Combined with results of mass spectrometry and bioinformatics analysis, we found that Toll4 possesses a binding peptide at TIR domain with wsv271 protein (Figure 4B and Figure S5A,B). In mammals, TLR4, a pattern recognition receptor, is tightly associated with the formation of neutrophil extracellular traps (Zhan et al. 2022; Clark et al. 2007). Therefore, immunoprecipitation assay was performed to confirm the interaction between wsv271 and Toll4 (Figure 4C). IF analysis also revealed that wsv271 and Toll4 protein were co‐localized in haemocytes of EV‐WSSV‐treated mud crabs (Figure 4D). Furthermore, we found that both mRNA and protein levels of Toll4 were significantly reduced when wsv271 was silenced (Figure 4E,F). To investigate whether Toll4 was involved in wsv271‐mediated P38‐MAPK pathway activation, EVs and Toll4‐siRNA were co‐injected into mud crabs (Figure S5C,D), followed by detection of P38‐MAPK pathway, the results showed that both mRNA and protein levels of ERK1 and ERK2 were significantly decreased when Toll4 was silenced (Figure 4G,H), moreover, the phosphorylation level of P38 was also decreased along with Toll4 silence (Figure 4I and Figure S5E), indicating that Toll4 was required for wsv271‐mediated P38‐MAPK pathway activation. Then, to demonstrate the effect of Toll4 on wsv271‐mediated NETosis‐like response, LPS (TLR4‐dependent P38‐MAPK pathway activator) was used (Fig. S5F), followed by detection of NETosis indicators. The results showed that the levels of MPO protein and MPO‐DNA complex were both significantly decreased when Toll4 was silenced, while increased when co‐injected with LPS (Figure 4J,K). Besides, IF analysis showed that histone‐H2A, MPO and NE1 proteins were distributed diffusely in haemocytes after treated with Toll4‐siRNA and LPS (Figure 4L‐N), the levels of ROS in haemocytes showed the same trend (Figure 4O). These data indicated that Toll4 was required for wsv271‐mediated NETosis‐like response. In addition, to explore whether Toll4 was relevant to wsv271‐mediated antiviral immune response, viral copy numbers were measured and the results showed that silencing of Toll4 significantly reduced the efficiency of EVs‐mediated viral suppression (Figure 4P), besides, the proportion of virus‐carrying cells was also decreased to 18.5% when Toll4 was silenced (Figure 4Q and Figure S5G). Taken together, the above data suggest that wsv271 activates P38‐MAPK pathway and further regulates NETosis‐like response and viral replication by interacting with Toll4.

### 3.5 Toll4 activates wsv271‐Dependent P38‐MAPK Pathway via Recruiting MyD88

**FIGURE 5:**
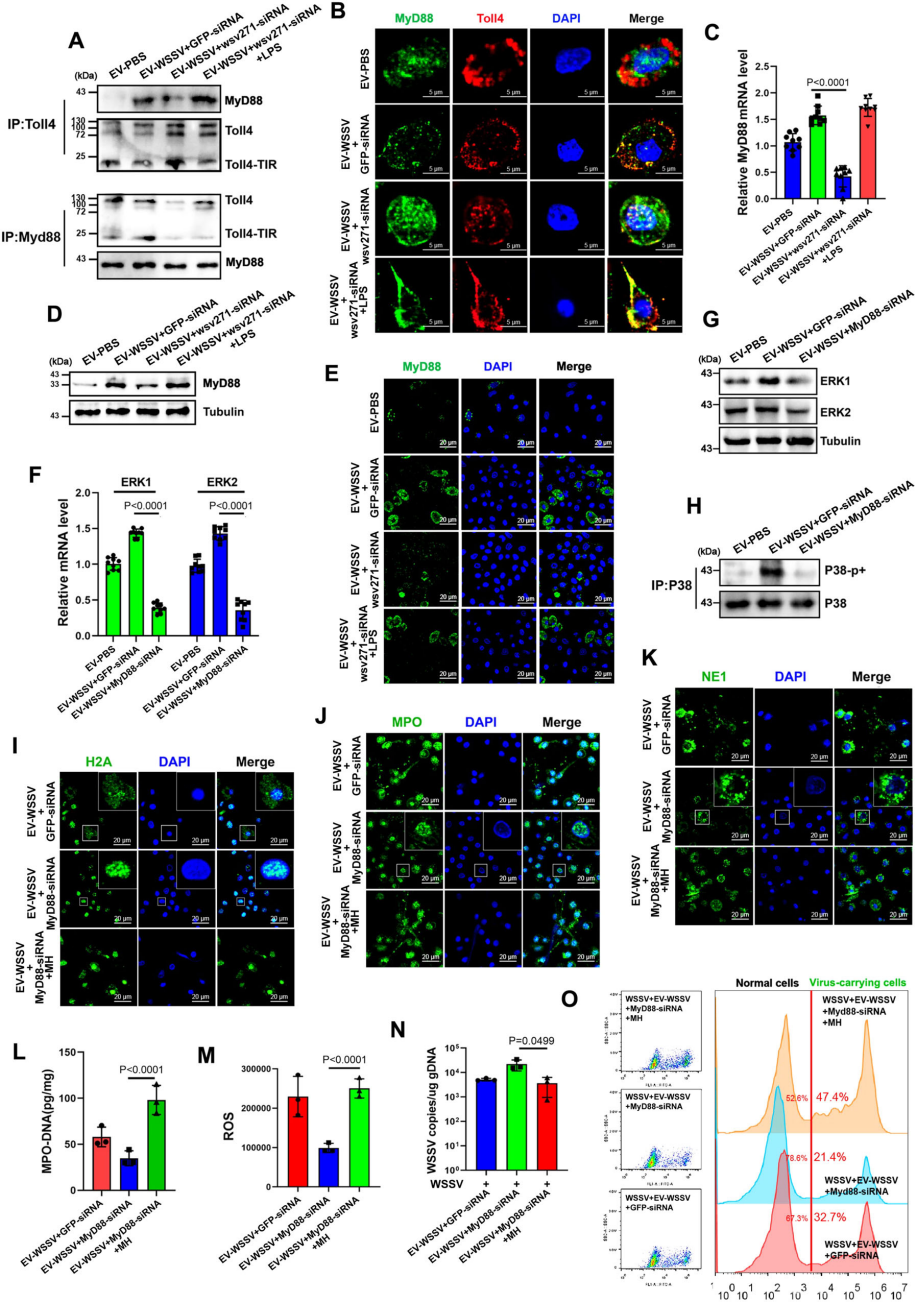
**Toll4 activates wsv271‐dependent P38‐MAPK pathway via recruiting MyD88. (A)** The interactions between Toll4 and MyD88 after treated with EVs, wsv271‐siRNA and LPS. Cell lysates were subjected to immunoprecipitation analysis with anti‐Toll4 IgG and anti‐MyD88 IgG, followed by Western blot using the indicated antibodies. **(B)** Localization of Toll4 and MyD88 protein in mud crab haemocytes after treated with EVs, wsv271‐siRNA and LPS were detected by IF, DAPI was used to stain the nucleus, scale bar, 5 µm. **(C–E)** RT‐qPCR, Western blot and IF were performed to detect the mRNA and protein levels of MyD88 in haemocytes after treated with EVs, wsv271‐siRNA and LPS, tubulin was used as the internal control, the nucleus was stained with DAPI, scale bar, 50 µm. **(F–G)** RT‐qPCR and Western blot were performed to detect the mRNA and protein levels of ERK1 and ERK2 in haemocytes after treated with EVs and MyD88‐siRNA, tubulin was used as the internal control. **(H)** Western blot assay was performed to detect the phosphorylation level of P38 protein after treated with EVs and MyD88‐siRNA, immunoprecipitated P38 was served as the internal control. **(I–K)** The cellular localization of histone‐H2A **(I)**, MPO **(J)** and NE1 **(K)** proteins in haemocytes after treated with EVs, MyD88‐siRNA and MH. DAPI was used to stain the nucleus, scale bar, 20 µm. **(L)** The levels of MPO‐DNA complex in haemocytes after treated with EVs, MyD88‐siRNA and MH were quantified by ELISA. **(M)** The ROS levels in haemocytes after treated with EVs, MyD88‐siRNA and MH were detected by microplate reader. **(N)** Virus copy numbers in WSSV‐challenged mud crab after treated with EVs, MyD88‐siRNA and MH were detected by RT‐qPCR. **(O)** The proportion of virus‐carrying haemocytes in WSSV‐challenged mud crabs after treated with EVs, MyD88‐siRNA and MH were detected by flow cytometry, SYBR Green was used to stain viral particles. Results are the mean ± SD of three experiments.

Studies have shown that MyD88 in TLR4 pathway is involved in the activation of P38‐MAPK pathway (Zhou et al. 2018). Based on this, the interaction between TLR4 and MyD88 was evaluated, the results showed that the amount of MyD88 binding with Toll4 was significantly increased after EV‐WSSV treatment, but decreased when wsv271 was silenced (Figure 5A), IF analysis also revealed that MyD88 and Toll4 protein were co‐localized in haemocytes of EV‐WSSV‐treated mud crabs (Figure 5B), besides, the mRNA and protein levels of MyD88 showed the same trend (Figure 5C‐E), indicating that wsv271 could promote the expression of MyD88 and facilitate the recruitment of MyD88 to Toll4. Then, to analysis whether MyD88 was involved in wsv271‐mediated P38‐MAPK pathway activation, MyD88 was silenced (Figure S6A,B), followed by detection of ERK1/ERK2 and the phosphorylation level of P38, the results showed that EVs‐mediated P38‐MAPK pathway activation was strongly suppressed when MyD88 was silenced (Figure 5F‐H). Next, to reveal the function of MyD88 on EVs‐mediated NETosis‐like response, the cellular localization of histone‐H2A, MPO and NE1 proteins, the levels of MPO protein and ROS were detected after co‐injection of EVs and MyD88‐siRNA. The results showed that MyD88 silence notably suppressed NETosis activated by EV‐WSSV (Figure 5I‐M). In addition, by detecting viral infection, we found that EVs‐mediated viral suppression was weakened when MyD88 was silenced (Figure 5N). The results of both flow cytometry and IF showed that MyD88 silence could also reduce the proportion of virus‐carrying cells (Figure 5O and Figure S6C). Taken together, the above data suggest that wsv271 can interact with Toll4 to recruit MyD88 and then activate P38‐MAPK pathway to regulate NETosis‐like response and viral replication.

### 3.6 P38‐Mediated PAD4 Phosphorylation and Nuclear Translocation Is Required for the Activation of NETosis‐Like Response

**FIGURE 6:**
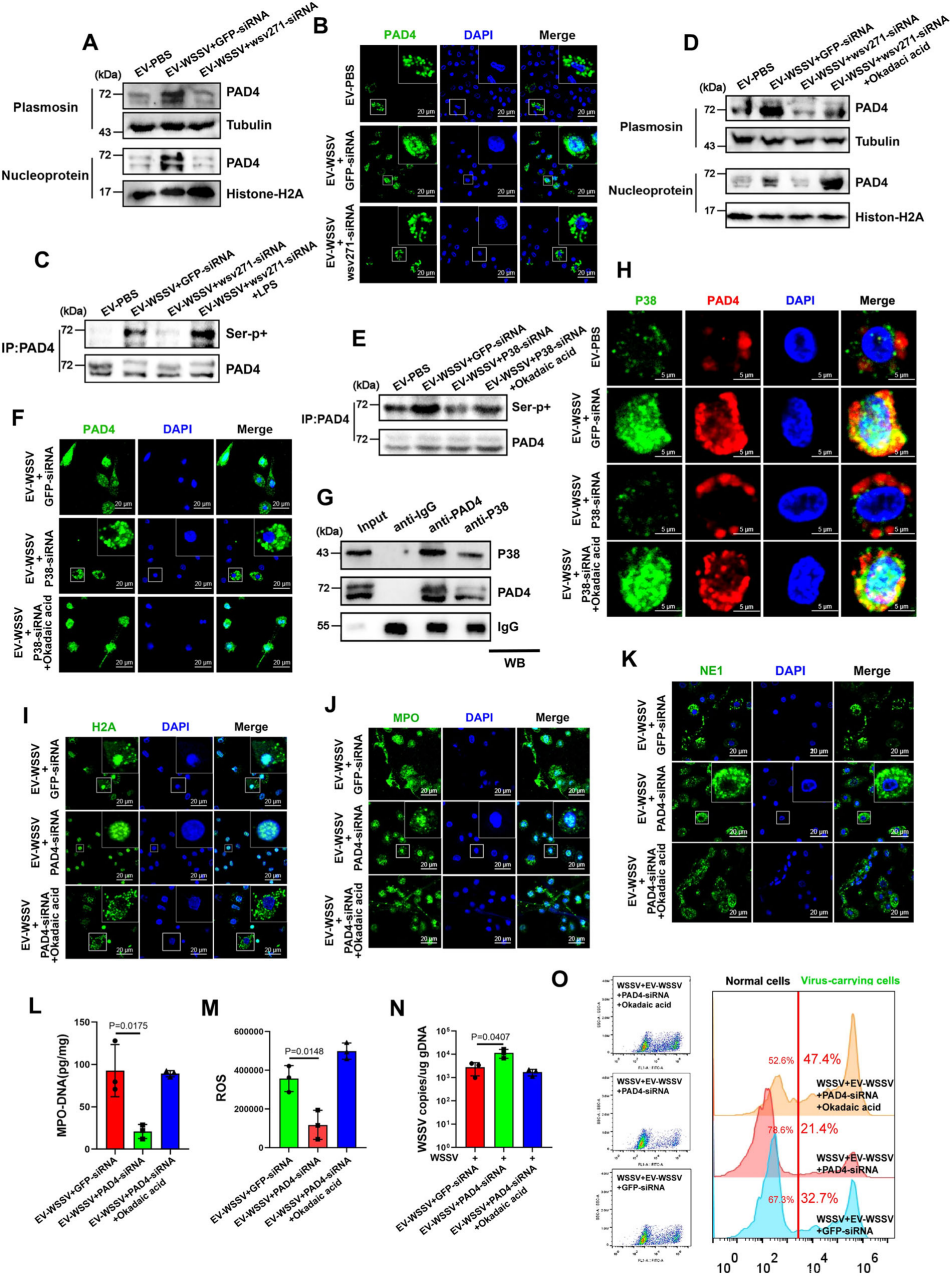
**P38‐mediated PAD4 phosphorylation and nuclear translocation is required for the activation of NETosis‐like response. (A)** The content of PAD4 protein in cytoplasm and nucleus after treated with EVs and wsv271‐siRNA were detected by Western blot, tubulin and histone‐H2A were used as cytoplasmic and nuclear reference proteins, respectively. **(B)** The cellular localization of PAD4 in haemocytes after treated with EVs and wsv271‐siRNA, DAPI was used to stain the nucleus, scale bar, 50 µm. **(C)** The phosphorylation levels of PAD4 protein after treated with EVs, wsv271‐siRNA and Okadaic acid (Phosphatase inhibitor) were detected by Western blot, immunoprecipitated PAD4 was served as the internal control. **(D)** The content of PAD4 protein in cytoplasm and nucleus after treated with EVs, wsv271‐siRNA and Okadaic acid. **(E and F)** The phosphorylation levels and cellular localization of PAD4 after treated with EVs, P38‐siRNA and Okadaic acid, immunoprecipitated P38 was served as the internal control, DAPI was used to stain the nucleus, scale bar, 50 µm. **(G)** The interactions between P38 and PAD4. Cell lysates were subjected to immunoprecipitation analysis with anti‐P38 IgG and anti‐PAD4 IgG, followed by Western blot using the indicated antibodies. **(H)** Localization of P38 and PAD4 protein in mud crab haemocytes after treated with EVs, wsv271‐siRNA and Okadaic acid were detected by IF, DAPI was used to stain the nucleus, scale bar, 5 µm. **(I–K)** The cellular localization of histone‐H2A **(I)**, MPO **(J)** and NE1 **(K)** proteins in haemocytes after treated with EVs, PAD4‐siRNA and Okadaic acid. DAPI was used to stain the nucleus, scale bar, 20 µm. **(L)** The levels of MPO‐DNA complex in haemocytes after treated with EVs, PAD4‐siRNA and Okadaic acid were quantified by ELISA. **(M)** The ROS levels in haemocytes after treated with EVs, PAD4‐siRNA and Okadaic acid were detected by microplate reader. **(N)** Virus copy numbers in WSSV‐challenged mud crab after treated with EVs, PAD4‐siRNA and Okadaic acid were detected by RT‐qPCR. **(O)** The proportion of virus‐carrying haemocytes in WSSV‐challenged mud crabs after treated with EVs, PAD4‐siRNA and Okadaic acid were detected by flow cytometry, SYBR Green was used to stain viral particles. Results are the mean ± SD of three experiments.

It is known that the key step for NETosis activation is the phosphorylation and nuclear translocation of PAD4 in vertebrates (Zhu et al. 2023). Based on this, IF and Western blot analyses were performed to detect the cellular distribution of PAD4. The results showed that the content of PAD4 protein was prominently increased in the nucleus after injection with EV‐WSSV, but this phenomenon was suppressed when wsv271 was silenced (Figure 6A,B). Then, through using phosphorylation antibodies and okadaic acid (phosphatase inhibitor), we found that PAD4 were hyperphosphorylated at serine site (Figure 6C), besides, it was found that phosphorylation modification could increase the content of PAD4 in the nucleus (Figure 6D and Figure S7A). P38 is a protein kinase and is phosphorylated during EVs‐mediated MAPK pathway activation, thus, whether PAD4 phosphorylation is regulated by P38 needs to be addressed. Interestingly, we found that the phosphorylation level of PAD4 was decreased along with P38 silence (Figure 6E), the nuclear translocation of PAD4 also showed the same trend (Figure 6F), besides, immunoprecipitation and IF analysis revealed the direct interaction between P38 and PAD4 proteins in haemocytes (Figure 6G,H), these data suggested that P38 could directly phosphorylate PAD4 and mediate its nuclear translocation.

Subsequently, the role of wsv271, Toll4 and MyD88 on the phosphorylation and nuclear translocation of PAD4 was detected. It was found that the phosphorylation and nuclear translocation of PAD4 was suppressed after knocking down wsv271, Toll4 and MyD88 (Figure S7B,C). Next, to investigate whether PAD4 participates in the EVs‐mediated NETosis‐like and antiviral immune response, PAD4‐siRNA and Okadaic acid were co‐injected into mud crabs (Fig. S7D and S7E). Through detecting NETosis indicators, including the cellular localization of histone‐H2A, MPO, and NE1 proteins, the levels of MPO‐DNA complexes and ROS, we found that PAD4 phosphorylation could facilitate EVs‐mediated NETosis‐like response (Figure 6I‐M). In addition, we found that PAD4 silence significantly reduced the efficiency of EVs‐mediated viral suppression (Figure 6N) and decreased the proportion of virus‐carrying cells to 21.4% (Figure 6O and Figure S7F). Taken together, the above data suggest that P38 in MAPK pathway can phosphorylate PAD4 to activate NETosis‐like response and further suppress viral infection.

### 3.7 Phosphorylated PAD4 Catalyze Citrullination of Histone‐H3 to Activate NETosis‐Like Response

**FIGURE 7:**
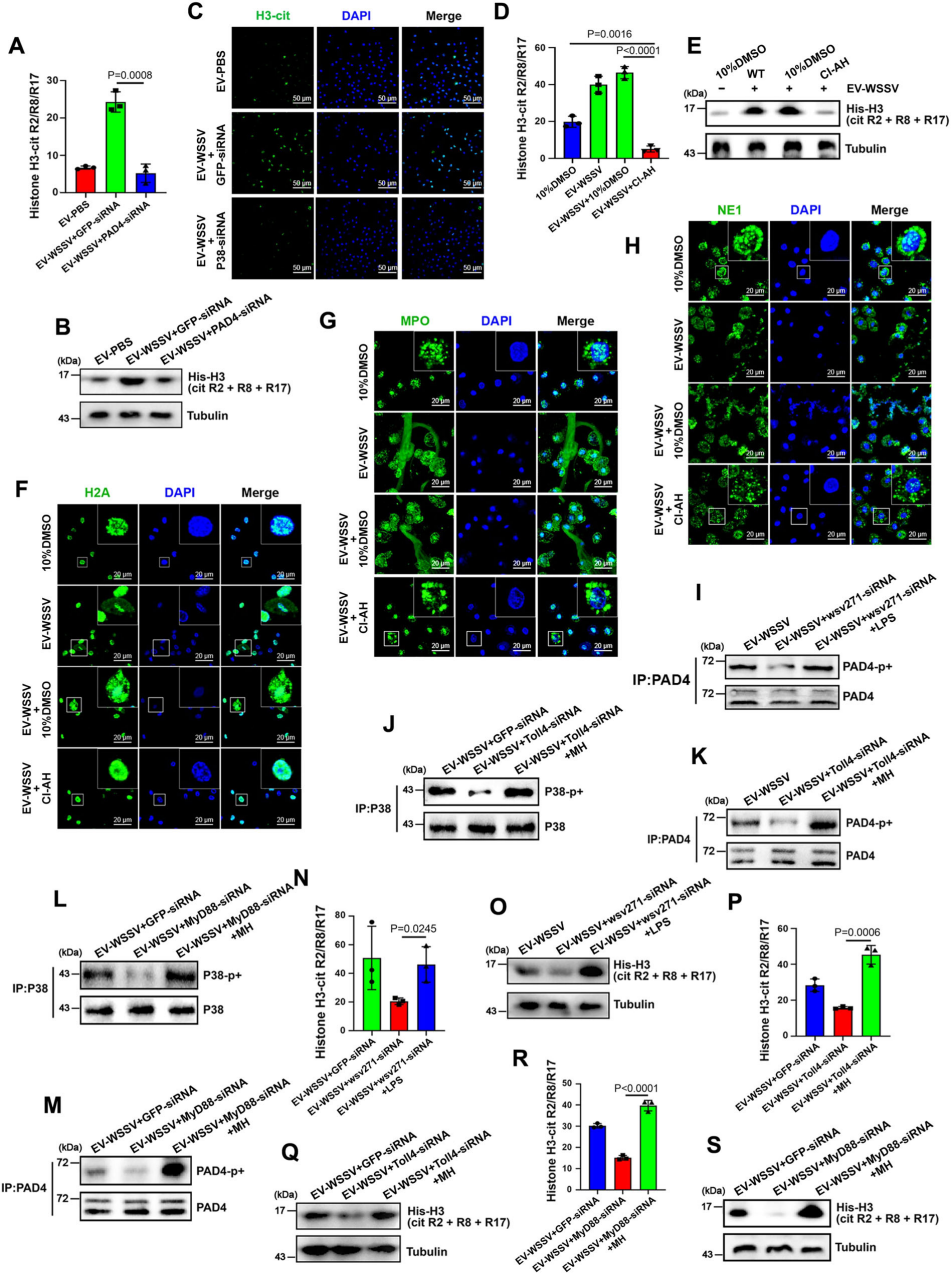
**Phosphorylated PAD4 catalyze citrullination of histone‐H3 to activate NETosis‐like response. (A–C)** The citrullination levels of histone‐H3 in haemocytes after treated with EVs and PAD4‐siRNA were detected by ELISA **(A)**, Western blot **(B)** and IF **(C)**, tubulin was used as the internal control, DAPI was used to stain the nucleus, scale bar, 50 µm. **(D and E)** The citrullination levels of histone‐H3 in haemocytes after treated with citrullination inhibitor CI‐amidine hydrochloride for 3 h were assessed by using ELISA **(D)** and Western blot **(E)**, 10% dimethyl sulfoxide (DMSO) solution was utilized as the negative control. **(F–H)** The cellular localization of histone‐H2A **(F)**, MPO **(G)** and NE1 **(H)** proteins in haemocytes after treated with EVs and CI‐AH. DAPI was used to stain the nucleus, scale bar, 20 µm. **(I)** The phosphorylation levels of PAD4 protein after treated with EVs, wsv271‐siRNA and LPS were detected by Western blot, immunoprecipitated PAD4 was served as the internal control. **(J and K)** The phosphorylation levels of P38 and PAD4 protein after treated with EVs, Toll4‐siRNA and MH were detected by Western blot, immunoprecipitated P38 and PAD4 were served as the internal control. **(L and M)** The phosphorylation levels of P38 and PAD4 protein after treated with EVs, MyD88‐siRNA and MH were detected by Western‐blot, immunoprecipitated P38 and PAD4 were served as the internal control. **(N and O)** The citrullination levels of histone‐H3 in haemocytes after treated with EVs, wsv271‐siRNA and LPS were detected by ELISA **(N)** and Western blot **(O)**, tubulin was used as the internal control. **(P and Q)** The citrullination levels of histone‐H3 in haemocytes after treated with EVs, Toll4‐siRNA and MH were detected by ELISA **(P)** and Western‐blot **(Q)**, tubulin was used as the internal control. **(R and S)** The citrullination levels of histone‐H3 in haemocytes after treated with EVs, MyD88‐siRNA and MH were detected by ELISA **(R)** and Western‐blot **(S)**, tubulin was used as the internal control. All data represented were the mean ± SD of at least three independent experiments.

PAD4‐mediated histone‐H3 citrullination is widely recognized as the NETosis‐triggered switch in vertebrates (Ronchetti et al. 2022). Based on this, we conducted ELISA, Western blotting and IF assays to assess the citrullination levels of histone H3. The results showed that the citrullination level of histone‐H3 was increased following EV‐WSSV treatment but strongly decreased when PAD4 was inhibited (Figure 7A‐C), indicating that PAD4 is essential for EVs‐mediated histone‐H3 citrullination in mud crabs. Then, to reveal the role of histone‐H3 citrullination in EVs‐mediated NETosis‐like response, histone citrullination inhibitor CI‐amidine hydrochloride was used (Figure 7D,E and Figure S8A–C). By analyzing NETosis indicators, including the cellular localization of histone‐H2A, MPO and NE1 proteins, we found that histone‐H3 citrullination is essential for EVs‐mediated NETosis‐like response (Figure 7F‐H). After that, to reveal the involvement of wsv271, Toll4 and P38‐MAPK pathway in this process, wsv271, Toll4 and MyD88 were knocked down, respectively, followed by detection of P38 and PAD4 phosphorylation and histone‐H3 citrullination. The results showed that wsv271, Toll4 and MyD88 could all facilitate the phosphorylation of P38 and PAD4 (Figure 7I‐M), and further citrullinate histone‐H3 to activate NETosis‐like response process (Figure 7N‐S and Figure S8D–F).

**FIGURE 8:**
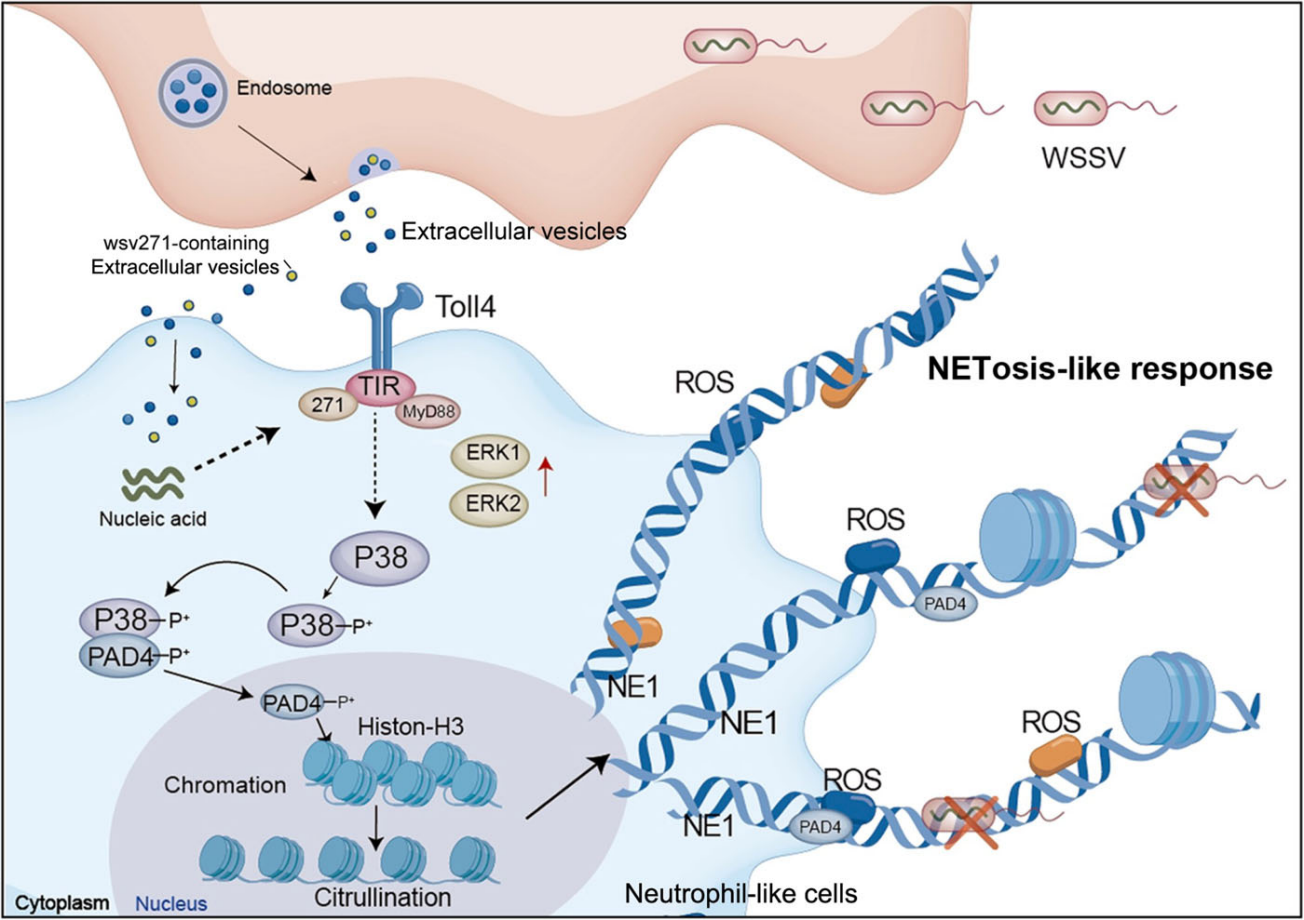
The proposed schematic diagram for NETosis‐like response induced by EVs‐derived viral nucleic acid delivery. During WSSV infection, viral mRNA wsv271 was packaged in EVs secreted by haemocytes and transmitted to the surrounding EVs‐recipient cells, followed by translation into viral protein, and then interacted with the TIR domain of Toll4 to recruit MyD88, so as to activate P38‐MAPK pathway and further facilitate PAD4 phosphorylation and nuclear translocation to citrullinate histone‐H3, which eventually suppressed the spread of viral infection by inducing NETosis‐like response in haemocytes.

In summary, the findings of this study indicated that during WSSV infection, viral mRNA wsv271 was specifically carried by EVs and transmitted to the surrounding EVs‐recipient cells, followed by translation into viral protein, and then interacted with the TIR domain of Toll4 to recruit MyD88 so as to activate P38‐MAPK pathway and further facilitate PAD4 phosphorylation and nuclear translocation to citrullinate histone‐H3, which eventually suppressed the spread of viral infection by inducing NETosis‐like response in haemocytes (Figure 8).

## 4 Discussion

As a novel, newly identified tool for genetic exchange between cells, EVs are capable of carrying specific bio‐cargos to cope with external stimuli (Meckes et al. 2010; Pérez et al. 2019). Based on this, the ‘Trojan exosome hypothesis’ during viral infection was put forward (Gould et al. 2003). The viral substance, even infectious virus particles, can be packaged by EVs and transmitted to the host cells to mediate non‐receptor‐dependent infection (Fang et al. 2007). During HCV (hepatitis C virus) infection, the full‐length replication‐competent viral RNAs can be packaged into EVs to mediate receptor‐independent infection of other susceptible cells (Deng et al. 2019). Moreover, it was found that EVs secreted from HIV‐1 (human immunodeficiency virus type‐1)‐infected macrophages contribute to virus transfer to the uninfected cells by packaging HIV‐1 virus particles (Kadiu et al. 2012). So far, almost all studies relevant to EVs‐derived viral component delivery are reported in higher animals, and the virus type is an RNA virus (Guo et al. 2016; Jopling et al. 2005). In this study, we found that WSSV‐specific nucleic acid wsv271 could be packaged by EVs to participate in the anti‐viral immune response of mud crabs. Our results reveal that the components of DNA viruses could be transmitted by EVs. In addition, it is worth mentioning that viral protein translated by wsv271 could interact with the intracellular TIR domain of Toll4 but not the extracellular domain to activate NETosis‐like response. This is a non‐classical and non‐extracellular way of activating pattern recognition receptors. Thus, we revealed a novel NETosis induction mechanism mediated by EVs‐derived viral nucleic acid delivery, which proved the diversity of EVs‐mediated viral immunity and its universality in animals.

As we know, PAD4 can mediate histone‐H3 citrullination and further lead to chromatin degradation and nucleus disintegration, which is required for the formation of NETs (Thiam et al. 2020; Lewis et al. 2015; Li et al. 2010). Although PAD4‐mediated NETosis induction has been extensively studied, it is still unknown how PAD4, as a cytoplasmic protein, enters the nucleus during the formation of NETs. Here, we found that when P38‐MAPK signalling pathway is activated, P38 can phosphorylate PAD4 at serine site to mediate its nuclear translocation, thus, this study reveals how PAD4 is translocated to nucleus during NETs formation. Overall, the induction mechanism of NETosis‐like response in mud crabs is relatively conservative compared with that in mammals (Rebak et al. 2024; Feys et al. 2024), which suggests that NETosis may be an ancient defence weapon, and this will provide ideas for exploring the function of NETosis‐like response in lower animals. Since researchers found that Hantavirus can induce neutrophils to release extracellular traps (Raftery et al. 2014), the correlation between NETosis and viral infection has received great attention (Zhu et al. 2018; Narayana Moorthy et al. 2013). It has been reported that the NETosis‐dependent inflammatory response can effectively eliminate the invading pathogens (Schauer et al. 2014; Veras et al. 2020). However, NETosis‐relevant studies in crustaceans are still scarce. In this study, we identified neutrophil‐like cells from the haemocytes of mud crabs based on single‐cell transcriptomics and observed nucleus disintegration in the haemocytes during WSSV infection, as well as the cellular localization of histone, MPO and NE1 proteins. In the shore crab, it was found that the citrullination levels of histones in haemolymph were increased during pathogen infection, and reticular‐like structure appeared in haemocytes (Coates et al. 2023). Thus, based on the findings in mud crab and shore crab, we have evidence that NETosis, a manner of programmed cell death, was also existed in invertebrates and possesses antiviral functions. These studies not only demonstrated the conservatism of NETosis regulatory pathway in animals but also provided novel cellular targets for the selection and breeding of disease‐resistant seedlings in the aquaculture industry.

The innate immune system evolved during long‐term natural selection, which is possessed by almost all bio‐organisms (Butcher et al. 1999; Banchereau and Steinman 1998). It was not until the emergence of specialized lymphoid organs and lymphocytes (T cells and B cells) in the circulatory system that the acquired immune system mainly based on specific humoral immunity and cellular immunity, was developed in vertebrates (Flajnik and Kasahara 2010). Studies have shown that CD8^+^ and CD4^+^ T cells could produce anti‐virus antibody to eliminate viral particles during acute vaccinia viral infection (Harrington et al. 2002). Besides, in the face of COVID‐19 infection, the specific proteins secreted by T cells play a decisive role in the elimination of viral particles, and this process has memorability and specificity (Long et al. 2020). Based on these findings, we can learn that apart from innate immunity, acquired immunity in vertebrates also serves as an essential tool to cope with viral infection and possesses the characteristics of memorability and specificity (Taniguchi et al. 2003). Given the existence of acquired immune system, whether the role of innate immune system is weakened or even degraded in vertebrates is widely concerned by immunologists (Kimbrell and Beutler 2001). In our view, this problem could be clarified to some extent by evaluating the immunological role of EVs‐derived viral nucleic acid delivery. In vertebrates, viruses could exploit EVs to transmit viral substances or even viral particles to escape the host's immune surveillance (Xia et al. 2023; Bukong et al. 2014; Fu et al. 2014; Wang et al. 2022). While in invertebrates, research, including this study, suggests that host cells could drive EVs to present viral components to activate the immune state of EVs‐recipient cells (Tassetto et al. 2017), this antiviral immune mechanism has also been reported in lower plants (López‐Gomóllon and Baulcombe 2022) (Figure S9). Therefore, since EVs‐dependent pathway serves as a crucial innate immune approach, we propose the following hypothesis: compared with vertebrates, the EVs‐mediated innate immune response in invertebrates and plants possesses higher clearance efficiency against viruses.

## Author Contributions

**Hang Hu**: investigation, writing – original draft, methodology, validation. **Chunyi Zhong**: investigation. **Xinshan Zhao**: investigation. **Cheng Yi**: investigation. **Yi Gong**: writing – review and editing, funding acquisition, data curation, conceptualization, project administration.

## Conflicts of Interest

The authors declare no conflicts of interest.

## Supporting information




**Supplementary Material**: jev270210‐sup‐0001‐Appendices.docx

**Supplementary Material**: jev270210‐sup‐0002‐SuppMatt.xlsx

**Supplementary Material**: jev270210‐sup‐0003‐SuppMatt1‐6.rar

## Data Availability

The data that supports the findings of this study are available in the supplementary material of this article
