# Supplementary material for: NETosis‐Like Response Triggered by Extracellular Vesicle (EV)‐Delivered Viral Nucleic Acid, a Novel Cellular Immune Mechanism in Crustacean
Source: J Extracell Vesicles. 2025 Dec 28;14(12):e70210. doi: 10.1002/jev2.70210 (PMC12745171; doi:10.1002/jev2.70210)
Supplement: Supplementary file 1 — Supplementary Material: jev270210‐sup‐0001‐Appendices.docx [file JEV2-14-e70210-s003.docx]

**SUPPLEMENTAL DATA**

**
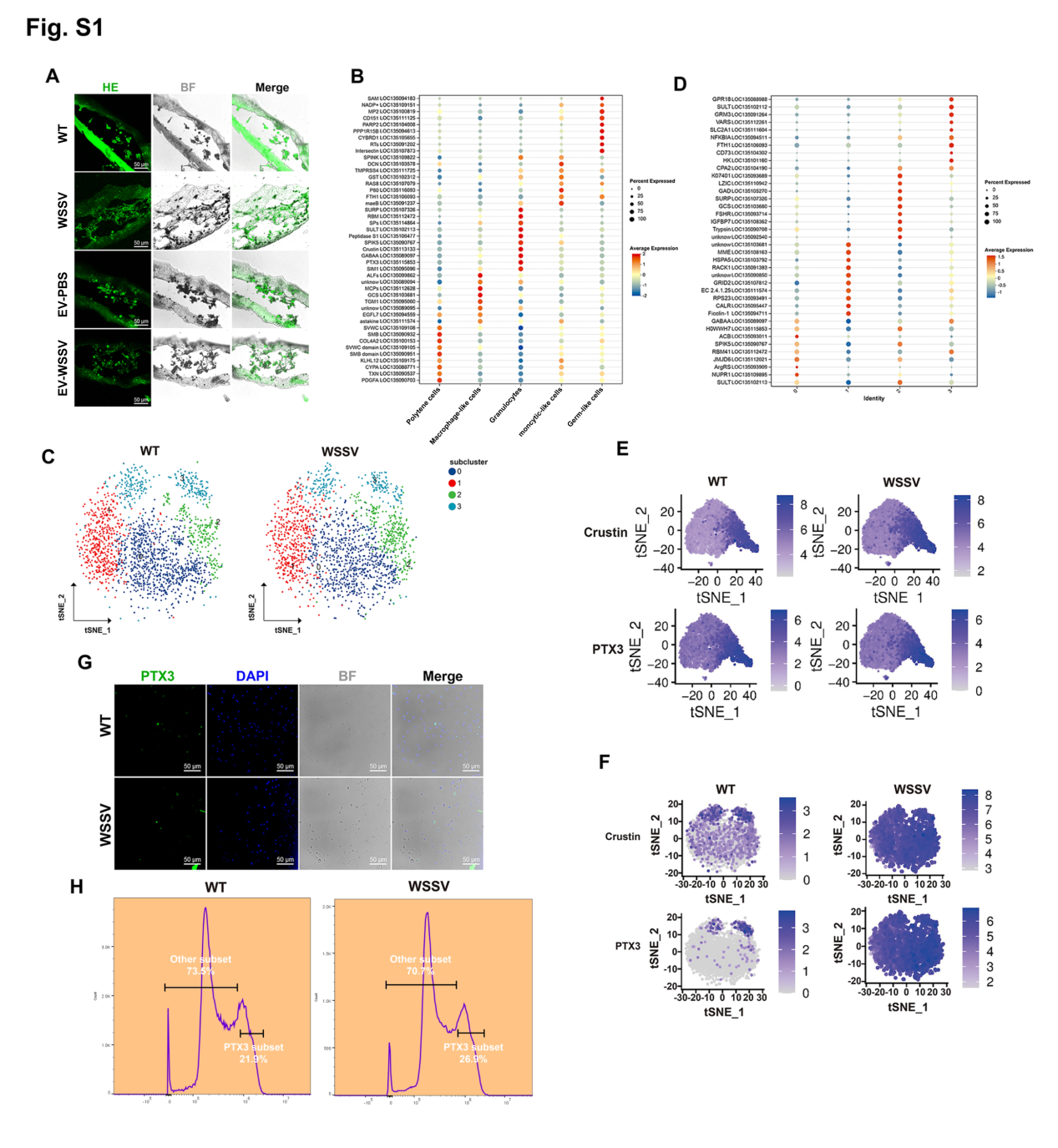
**

**Fig S1. (A)** Crab gill filaments were sectioned using paraffin and then stained with hematoxylin and eosin (HE). Followed by observation under an excitation wavelength of 488 nm. The thickness of the sections were 5 μm. **(B)** A bubble diagram showing ten representative marker genes for each major cluster. The gene name and its NCBI Gene ID is listed (left) and its expression level in each cell cluster is shown with different colors (right). **(C)** A t-SNE plot showing the distribution of subclusters in granulocytes from PBS-injection group and WSSV-injection group. **(D)** A bubble diagram showing ten representative marker genes for each subcluster. The gene name and its NCBI GeneID is listed (left) and its expression level in each cell is shown with different colors (right). **(E)** A t-SNE plot showing the distribution of Crustin and PTX3 genes in mud crab hemocytes from PBS-injection group and WSSV-injection group. **(F)** A t-SNE plot showing the distribution of Crustin and PTX3 genes in granulocytes from PBS-injection group and WSSV-injection group. **(I)** Confocal microscopy was employed to observe the hemocytes labeled with PTX3 antibody, the excitation wavelength utilized was 488 nm. DAPI was used to stain the nucleus. The scale bar is 50 μm. **(J)** Flow cytometry analysis was performed on hemocytes stained with FITC-conjugated anti-PTX3 antibody. In the WT group, 21.9% of the cells were FITC-positive, whereas in the WSSV-infected group, 26.8% of the cells were FITC-positive.


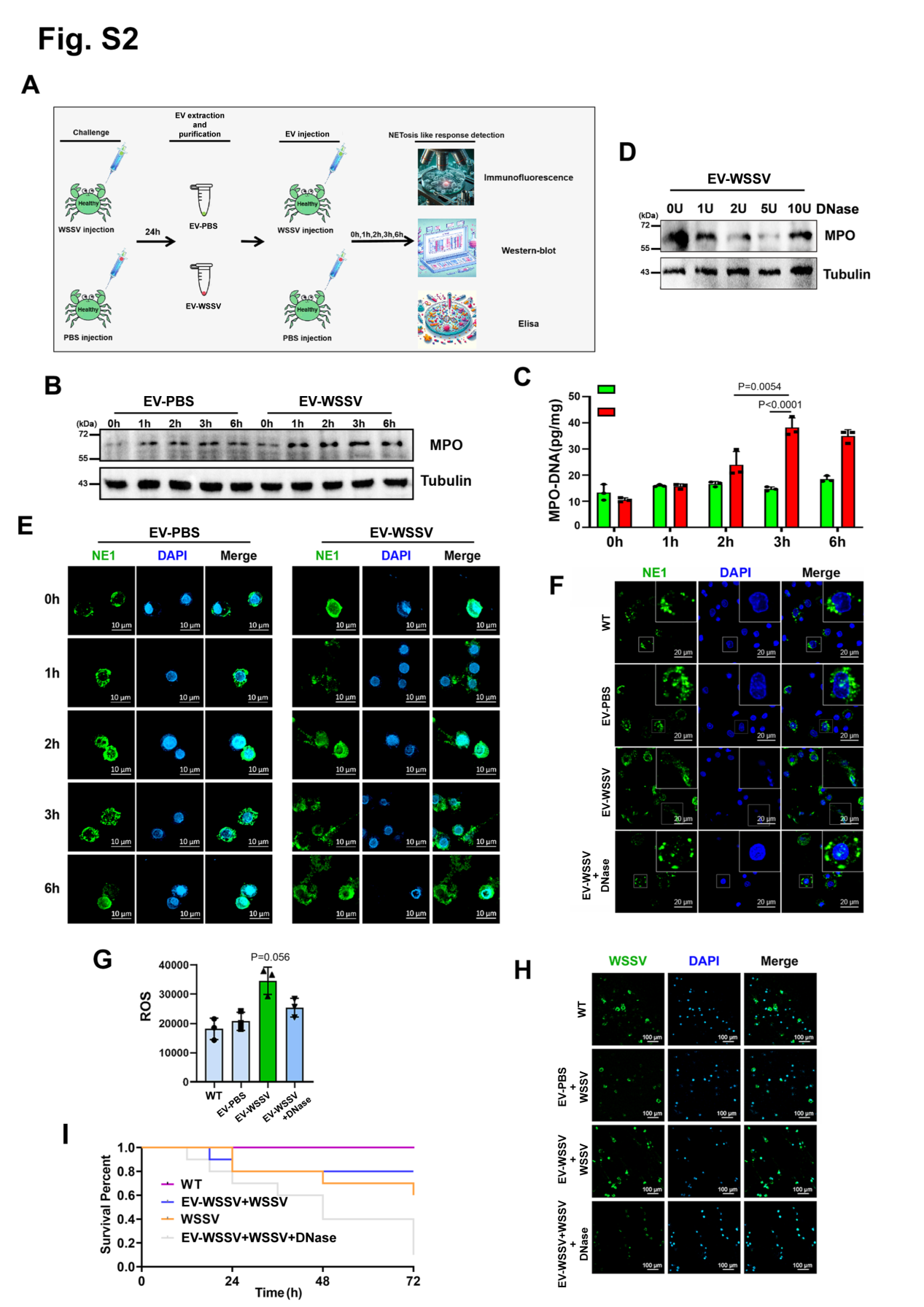


**Fig S2. (A)** Experimental procedures for detecting NETosis-related indicators. **(B)** The protein expression of MPO at different time points after EV-PBS or EV-WSSV injection was detected via Western blotting. Tubulin was used as an internal reference protein. **(C)** The content of the MPO-DNA complex at different time points after EV-PBS or EV-WSSV injection was determined via ELISA. **(D)** Western blotting analysis was performed to detect the expression of the MPO protein after injection of 0 U, 1 U, 2 U, 5 U, or 10 U of DNase under the premise of EV-WSSV. Tubulin was used as a reference protein. **(E)** The localization of NE1 protein at different time points after EV-PBS and EV-WSSV injection was detected by IF, and DAPI was used to stain the nuclei; scale bar, 10 μm. **(F)** The cellular localization of NE1 protein in hemocytes after treated with EVs and DNase, DAPI was used to stain the nucleus, scale bar, 20 μm. **(G)** The ROS levels in hemocytes after treated with EVs and DNase were detected by microplate reader. **(H)** The proportion of virus-carrying haemocytes in WSSV-challenged mud crabs after treatment with EVs and DNase was determined via fluorescence microscopy. SYBR Green was used to stain the viral particles; scale bar, 100 μm. **(I)** The survival rate analysis in mud crab after treated with WSSV, EV-WSSV+WSSV, EV-WSSV+WSSV+DNase. The mortality rate was recorded every 24 hours, with ten crabs examined in each group. The data are presented as the means ± S.D.s of three replicate experiments.


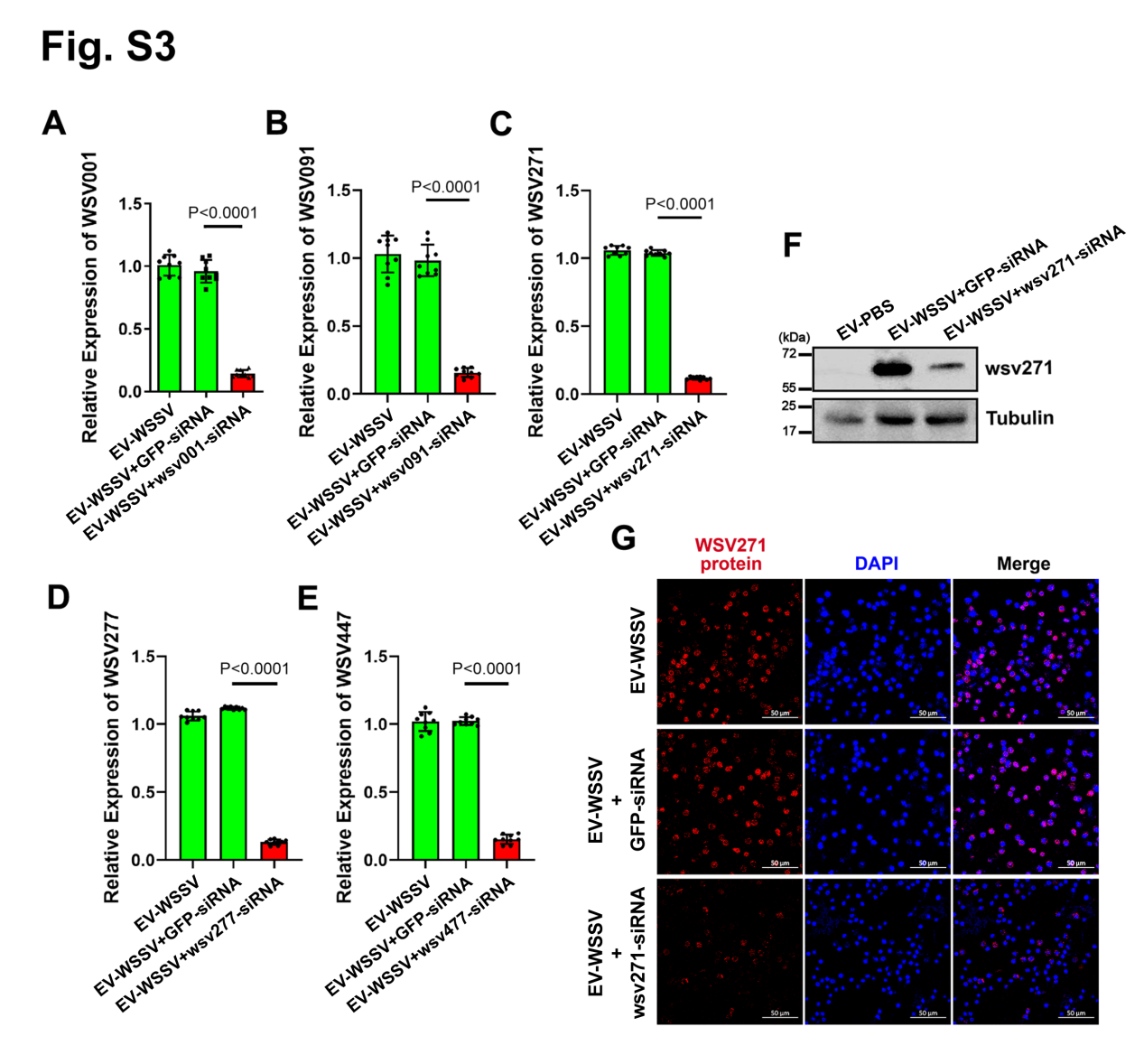


**Fig S3. (A-E)** RT-qPCR was used to detect the RNA interference efficiency of wsv001-siRNA, wsv091-siRNA, wsv271-siRNA, wsv277-siRNA and wsv447-siRNA in mud crabs after treatment with EVs. **(F-G)** The interference efficiency of wsv271 at the protein level in mud crabs after treatment with EVs was measured by Western blotting **(F)** and IF **(G)** analyses. Tubulin was used as the reference protein, and DAPI was used to stain the nucleus. The data are presented as the means ± S.D.s of three replicate experiments.


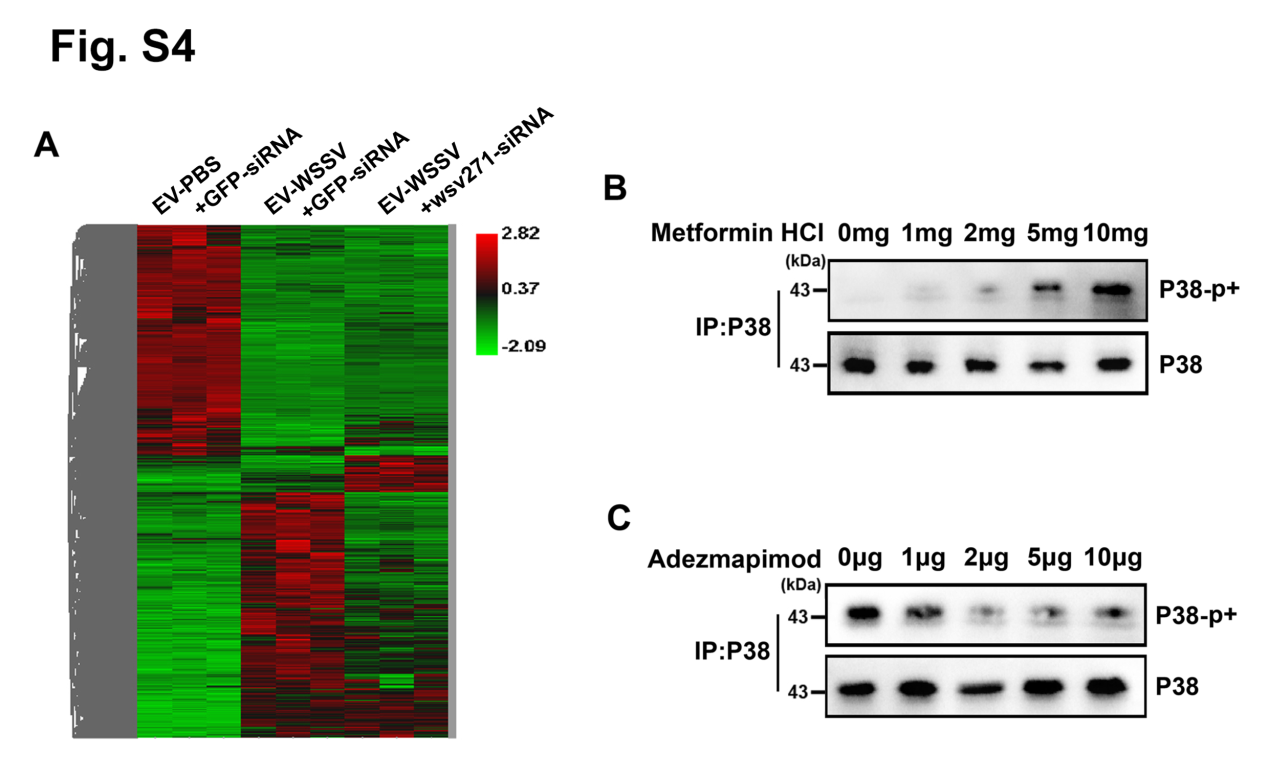


**Fig S4. (A)** Heatmap of transcriptomic sequencing data from mud crab haemocytes after treated with EV-PBS, EV-WSSV and EV-WSSV+wsv271-siRNA. **(B)** Western blotting was performed to determine the phosphorylation level of P38 after metformin HCL (a P38-MAPK pathway activator) injection at different doses in mud crabs. P38 protein served as the control. **(C)** Western blotting was performed to determine the phosphorylation level of P38 after the injection of different doses of adezmapimod (a P38-MAPK pathway inhibitor) into mud crabs.


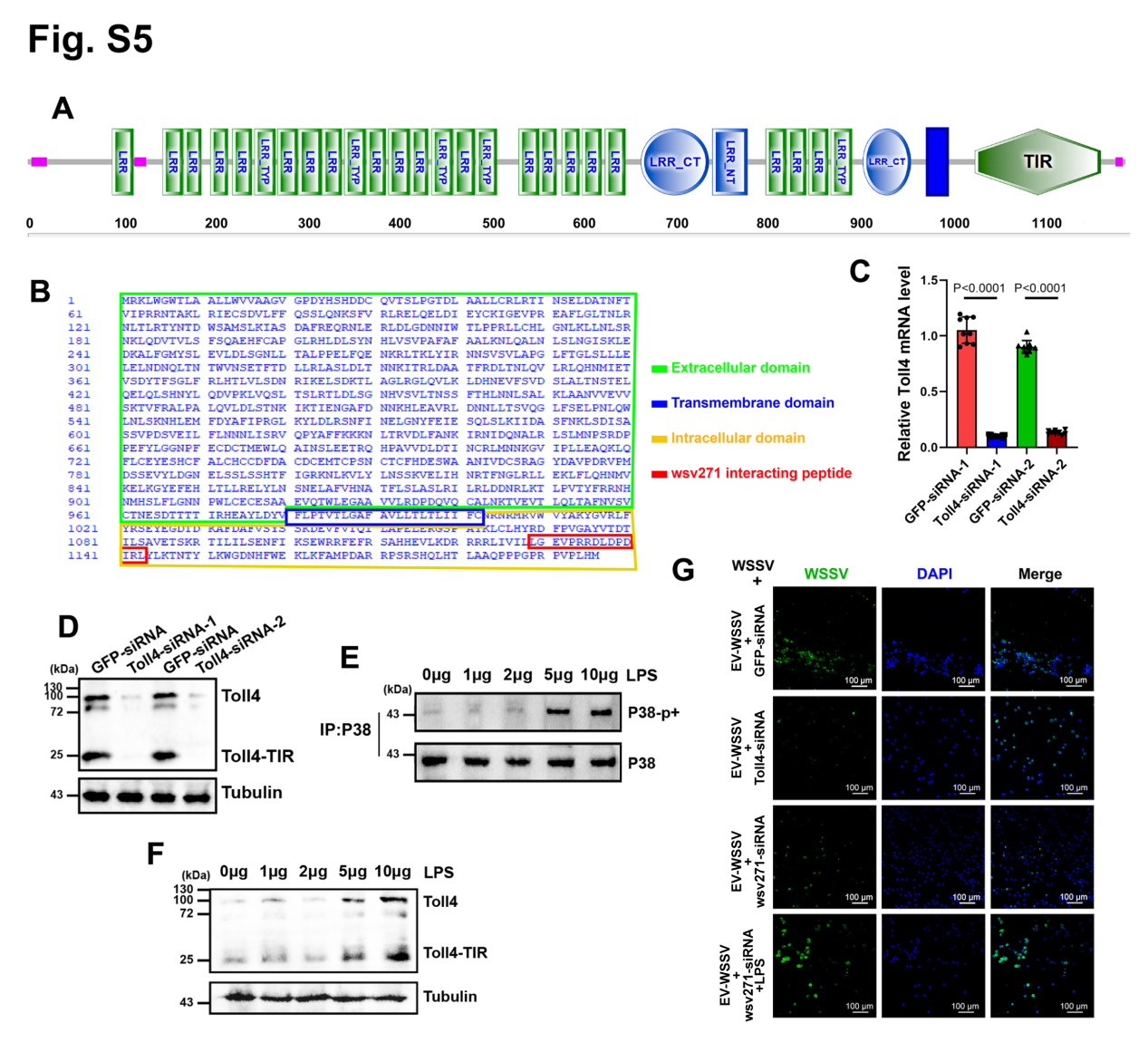


**Fig S5. (A)** Secondary structure of the Toll4 protein. **(B)** Sequence analysis of the Toll4 protein. The extracellular domain is marked in green, the transmembrane domain is marked in blue, the intracellular domain is marked in orange, and the wsv271-interacting peptide is marked in red. **(C-D)** RNAi efficiency detection of Toll4. Mud crabs were injected with siRNAs targeting Toll4, after which both the mRNA and protein expression levels of Toll4 were detected via RT‒qPCR **(C)** and Western blotting **(D)**, respectively. **(E)** Western blotting was used to detect the effect of LPS on P38 phosphorylation. P38 protein was used as a control. **(F)** Western blotting was used to detect the effect of LPS on the protein expression of Toll4. Tubulin was used as the reference protein. **(G)** The proportions of virus-carrying haemocytes in WSSV-challenged mud crabs after treatment with EVs, wsv271-siRNA, Toll4-siRNA and LPS were determined via fluorescence microscopy. SYBR Green was used to stain the viral particles; scale bar, 100 μm. The data are presented as the means ± S.D.s of three replicate experiments.


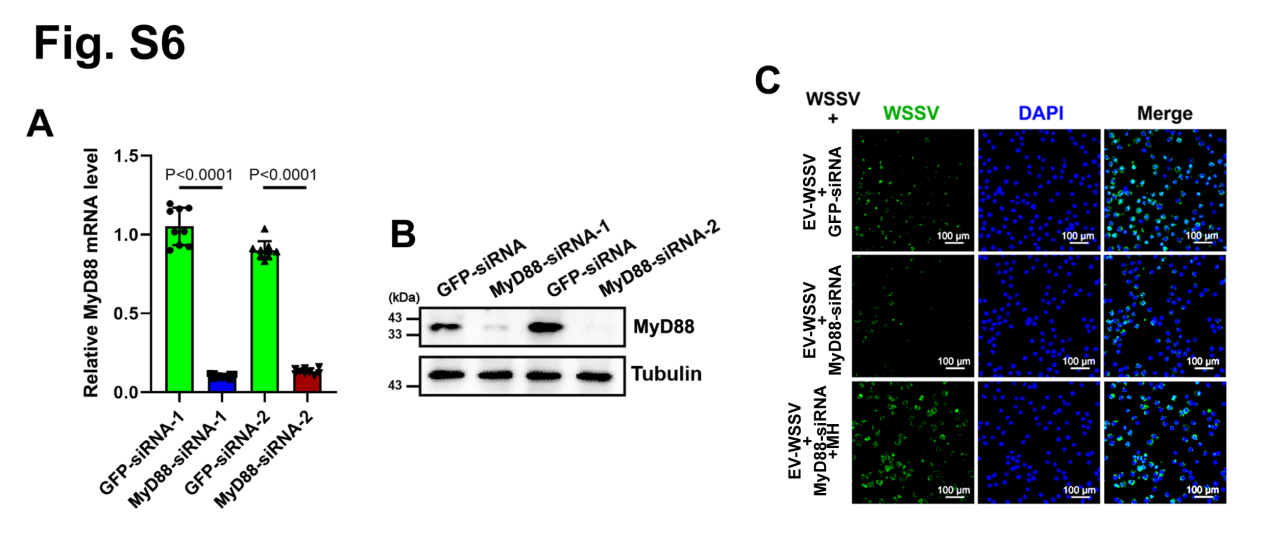


**Fig S6.** **(A-B)** RNAi efficiency detection of MyD88. Mud crabs were injected with siRNAs targeting MyD88, after which both the mRNA and protein expression levels of MyD88 were detected via RT‒qPCR **(A)** and Western blotting **(B),** respectively. **(C)** The proportions of virus-carrying haemocytes in WSSV-challenged mud crabs after treatment with EVs, MyD88-siRNA and MH were observed via fluorescence microscopy, and SYBR Green was used to stain the viral particles; scale bar, 100 μm. The data are presented as the means ± S.D.s of three replicate experiments.


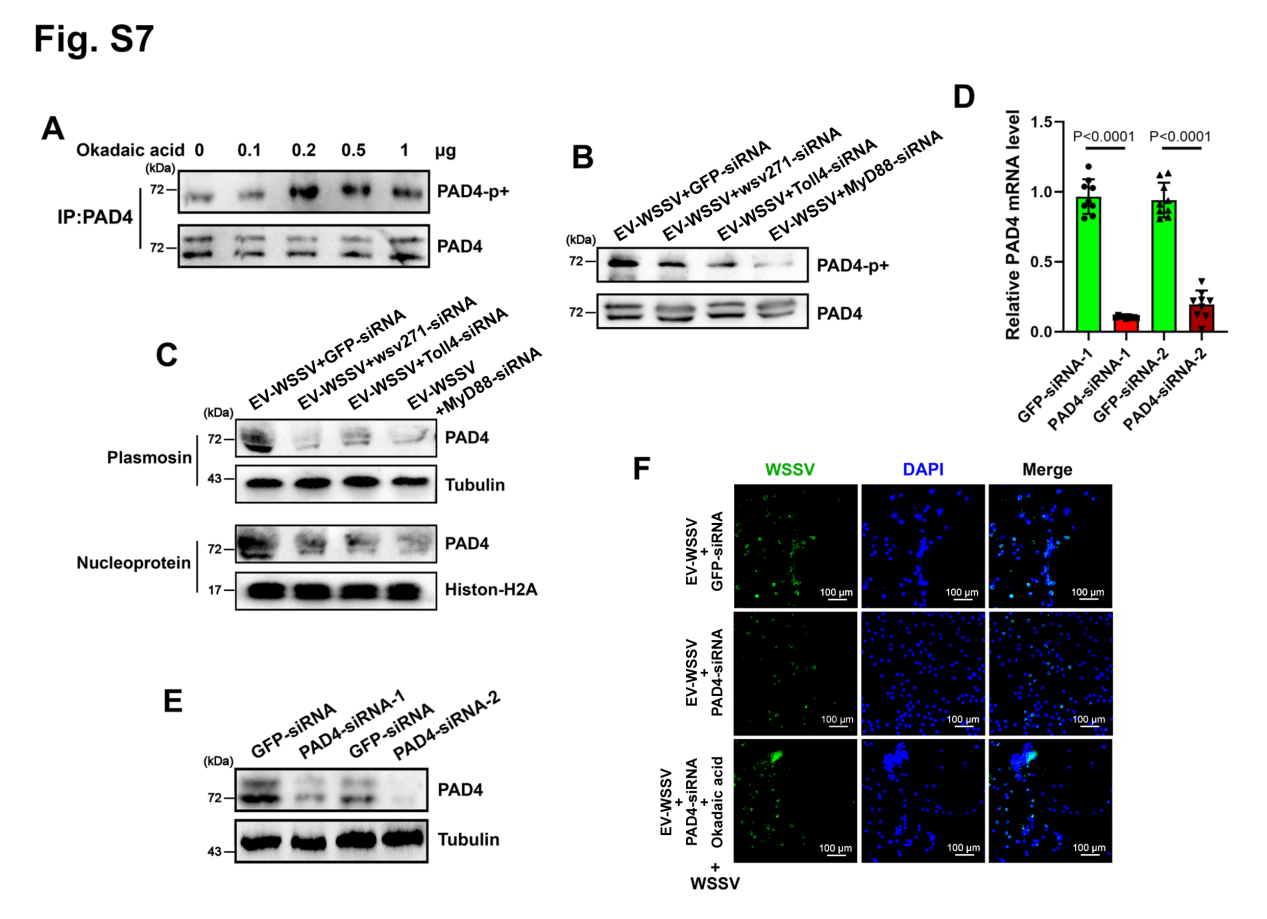


**Fig S7. (A)** Western blotting was performed to analyse the effects of okadaic acid on the phosphorylation level of the PAD4 protein. The PAD4 protein served as the control. **(B)** Effects of wsv271, Toll4 and MyD88 silencing on EVs-mediated PAD4 phosphorylation. **(C)** Western blotting was performed to determine the cytoplasmic and cellular expression levels of PAD4 after the indicated treatments. Tubulin and histone H2A were used as the cytoplasmic and nuclear proteins, respectively. **(D-E)** RNAi efficiency detection of PAD4. Mud crabs were injected with siRNAs targeting PAD4, after which both the mRNA and protein expression levels of PAD4 were detected via RT‒qPCR **(D)** and Western blotting **(E)**, respectively. **(F)** The proportions of virus-carrying haemocytes in WSSV-challenged mud crabs after treatment with EVs, PAD4-siRNA or okadaic acid were observed via fluorescence microscopy, and SYBR Green was used to stain the viral particles; scale bar, 100 μm. The data are presented as the means ± S.D.s of three replicate experiments.


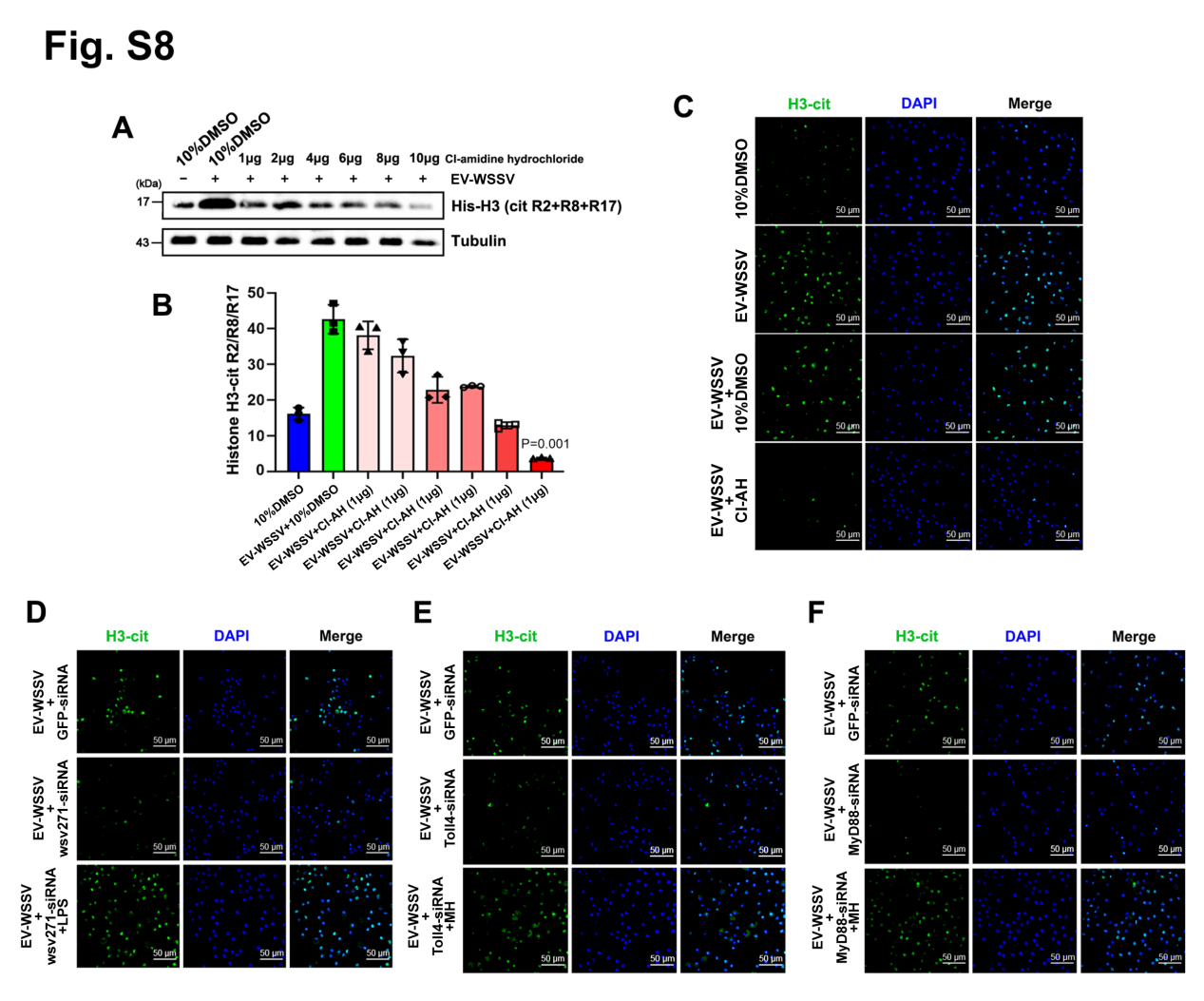


**Fig S8. (A-C)** The citrullination levels of Histone-H3 in the haemocytes of mud crabs after treatment with the citrullination inhibitor CI-amidine hydrochloride for 3 hours were assessed via Western blotting **(A)**, ELISA **(B)** and IF **(C)**. The concentration gradient of CI-AH was 0-10 µg, and 10% dimethyl sulfoxide (DMSO) solution was used as the negative control. **(D-E)** The citrullination levels of Histone-H3 in the hemocyte of mud crabs after treatment with EVs, wsv271-siRNA **(D)**, Toll4-siRNA **(E)**, MyD88-siRNA **(F)**, LPS and MH were detected by IF. DAPI was used to stain the nucleus; scale bar, 50 μm. The data are presented as the means ± S.D.s of three replicate experiments.


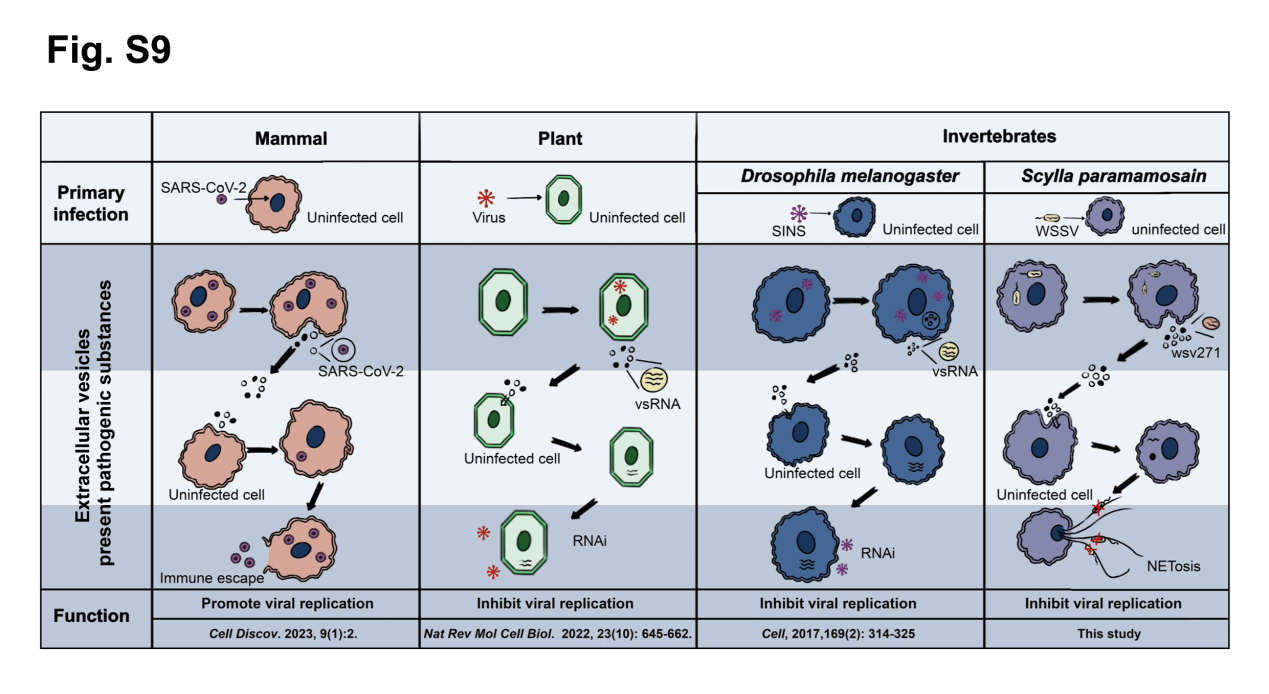


**Fig S9. The role of EVs-derived viral nucleic acid delivery in the antiviral innate immune response of vertebrates and invertebrates**. In mammal and plant, virus could exploit EVs to transmit viral particles or genomic RNA, so as to escape the host’s immune surveillance. While in invertebrates, results including this study reveal that host cells could drive EVs to transmit viral substances, and further activate the immune state of surrounding EVs-recipient cells to resist virus invasion.

**Table S1: Primers used in this study.**

| **Primer Primer sequence（5′- 3′） Usage** | | |  |
| --- | --- | --- | --- |
| Q-WSV001F | ATCGGGTGTTTTTGTGG | qRT-PCR |  |
| Q-WSV001R | GGTGTTTTTGTGGGCTA | qRT-PCR |  |
| Q-WSV091F | GATGTTCGCCCTTATGA | qRT-PCR |  |
| Q-WSV091R | TTGAGCGGAAGTTGATG | qRT-PCR |  |
| Q-WSV271F | TGAACTCATTCCCTTCC | qRT-PCR |  |
| Q-WSV271R | CGATAGATATAGCCGTTTT | qRT-PCR |  |
| Q-WSV277F | TTTGGCTCAGGAGAGAG | qRT-PCR |  |
| Q-WSV277R | TTTTGTTGGCAAGATTG | qRT-PCR |  |
| Q-WSV447F | TACCCAGGATGCGTATG | qRT-PCR |  |
| Q-WSV447R | TACCCAGGATGCGTATG | qRT-PCR |  |
| Q-P38-F | TCTACCAGGTGTTACGAGGGCT | qRT-PCR |  |
| Q-P38-R | CCGGGCCAGTCCAAAATCT | qRT-PCR |  |
| Q-β-actin-F | GCGGCAGTGGTCATCTCCT | qRT-PCR |  |
| Q-β-actin-R | GCCCTTCCTCACGCTATCCT | qRT-PCR |  |
| Q-ERK1-F | TGGTTGGGCTAAAAAAG | qRT-PCR |  |
| Q-ERK1-R | GGATGAGGCAAAGGTGT | qRT-PCR |  |
| Q-ERK2-F | CAAGTGTCTGGGTGTTTT | qRT-PCR |  |
| Q-ERK2-R | AATGACTCCTGACCGTTT | qRT-PCR | |
| Q-PAD4-F | CTGGGGAGAAGACTGCT | qRT-PCR | |
| Q-PAD4-R | AAGAGAGGGTGTAGGTGG | qRT-PCR | |
| Q-MyD88-F | TGAGCCCATCCACAAGA | qRT-PCR | |
| Q-MyD88-R | GCTGCCCACATCCAACT | qRT-PCR | |
| Q-WSSV1F | TTGGTTTCATGCCC GAGATT | WSSV Copies | |
| Q-WSSV1R | CCTTGGTCAGCCCCTTGA | WSSV Copies | |
| WSSV Probe | FAM- TGCTGCCGTCTCCAA-TAMRA | WSSV Copies | |
| WSV271 probe | FAM-CAATATCTCTGGCCGTATCCAC-FAM | FISH | |
| ERK1-1-1 | GATCACTAATACGACTCACTATAGGGAGCAGTGGCCTAGTGGTCAGCTTTATT | RNAi | |
| ERK1-1-2 | AATAAAGCTGACCACTAGGCCACTGCTCCCTATAGTGAGTCGTATTAGTGATC | RNAi |  |
| ERK1-1-3 | GATCACTAATACGACTCACTATAGGGTAAAGCTGACCACTAGGCCACTGCTTT | RNAi |  |
| ERK1-1-4 | AAAGCAGTGGCCTAGTGGTCAGCTTTACCCTATAGTGAGTCGTATTAGTGATC | RNAi |  |
| ERK1-2-1 | GATCACTAATACGACTCACTATAGGGGAGCCTCTGTAAGGGCATCACTAAATT | RNAi |  |
| ERK1-2-2 | AATTTAGTGATGCCCTTACAGAGGCTCCCCTATAGTGAGTCGTATTAGTGATC | RNAi |  |
| ERK1-2-3 | GATCACTAATACGACTCACTATAGGGTTTAGTGATGCCCTTACAGAGGCTCTT | RNAi |  |
| ERK1-2-4 | AAGAGCCTCTGTAAGGGCATCACTAAACCCTATAGTGAGTCGTATTAGTGATC | RNAi |  |
| ERK2-1-1 | GATCACTAATACGACTCACTATAGGGGAGGCGGCTTATACCTGGCCTTTATTT | RNAi |  |
| ERK2-1-2 | AAATAAAGGCCAGGTATAAGCCGCCTCCCCTATAGTGAGTCGTATTAGTGATC | RNAi |  |
| ERK2-1-3 | GATCACTAATACGACTCACTATAGGGATAAAGGCCAGGTATAAGCCGCCTCTT | RNAi |  |
| ERK2-1-4 | AAGAGGCGGCTTATACCTGGCCTTTATCCCTATAGTGAGTCGTATTAGTGATC | RNAi |  |
| ERK2-2-1 | GATCACTAATACGACTCACTATAGGGTGGCCACTGGTATTAGGCTATTATTTT | RNAi |  |
| ERK2-2-2 | AAAATAATAGCCTAATACCAGTGGCCACCCTATAGTGAGTCGTATTAGTGATC | RNAi |  |
| ERK2-2-3 | GATCACTAATACGACTCACTATAGGGAATAATAGCCTAATACCAGTGGCCATT | RNAi |  |
| ERK2-2-4 | AATGGCCACTGGTATTAGGCTATTATTCCCTATAGTGAGTCGTATTAGTGATC | RNAi |  |
| MyD88-1-1 | GATCACTAATACGACTCACTATAGGGCAGTCGGCTATAACATGGAATATAATT | RNAi |  |
| MyD88-1-2 | AATTCTCTTCCATGTTATAGCCGACTGCCCTATAGTGAGTCGTATTAGTGATC | RNAi |  |
| MyD88-1-3 | GATCACTAATACGACTCACTATAGGGTTCTCTTCCATGTTATAGCCGACTGTT | RNAi |  |
| MyD88-1-4 | AACAGTCGGCTATAACATGGAATATAACCCTATAGTGAGTCGTATTAGTGATC | RNAi |  |
| MyD88-2-1 | GATCACTAATACGACTCACTATAGGGGAGTTGGATGTGGGCAGCATCAGTATT | RNAi |  |
| MyD88-2-2 | AATACTGATGCTGCCCACATCCAACTCCCCTATAGTGAGTCGTATTAGTGATC | RNAi |  |
| MyD88-2-3 | GATCACTAATACGACTCACTATAGGGTACTGATGCTGCCCACATCCAACTCTT | RNAi |  |
| MyD88-2-4 | AAGAGTTGGATGTGGGCAGCATCAGTACCCTATAGTGAGTCGTATTAGTGATC | RNAi |  |
| P38-1-1 | GATCACTAATACGACTCACTATAGGGCACAGAGTCAGAAATGACAGGTTATTT | RNAi |  |
| P38-1-2 | AAATAACCTGTCATTTCTGACTCTGTGCCCTATAGTGAGTCGTATTAGTGATC | RNAi |  |
| P38-1-3 | GATCACTAATACGACTCACTATAGGGATAACCTGTCATTTCTGACTCTGTGTT | RNAi |  |
| P38-1-4 | AACACAGAGTCAGAAATGACAGGTTATCCCTATAGTGAGTCGTATTAGTGATC | RNAi |  |
| P38-2-1 | GATCACTAATACGACTCACTATAGGGCGCCCAGGCAGGATCTTGTTGATAATT | RNAi |  |
| P38-2-2 | AATTATCAACAAGATCCTGCCTGGGCGCCCCTATAGTGAGTCGTATTAGTGATC | RNAi |  |
| P38-2-3 | GATCACTAATACGACTCACTATAGGGTTATCAACAAGATCCTGCCTGGGCGCTT | RNAi |  |
| P38-2-4 | AACGCCCAGGCAGGATCTTGTTGATAACCCTATAGTGAGTCGTATTAGTGATC | RNAi |  |
| PAD4-1-1 | GATCACTAATACGACTCACTATAGGGGCAGCGCACATGAGCTTGATATTAATT | RNAi |  |
| PAD4-1-2 | AATTAATATCAAGCTCATGTGCGCTGCCCCTATAGTGAGTCGTATTAGTGATC | RNAi |  |
| PAD4-1-3 | GATCACTAATACGACTCACTATAGGGTTAATATCAAGCTCATGTGCGCTGCTT | RNAi |  |
| PAD4-1-4 | AAGCAGCGCACATGAGCTTGATATTAACCCTATAGTGAGTCGTATTAGTGATC | RNAi |  |
| PAD4-2-1 | GATCACTAATACGACTCACTATAGGGCAGCGCACATGAGCTTGATATTAAATT | RNAi |  |
| PAD4-2-2 | AATTTATATCAAGCTCATGTGGCTGCCCTATAGTGAGTCGTATTAGTGATC | RNAi |  |
| PAD4-2-3 | GATCACTAATACGACTCACTATAGGGTTTATATCAAGCTCATGTGGCTGTT | RNAi |  |
| PAD4-2-4 | AACAGCGCACATGAGCTTGATATTAAACCCTATAGTGAGTCGTATTAGTGATC | RNAi |  |
| siGFP-1 | GATCACTAATACGACTCACTATAGGGGGAGTTGTCCCAATTCTTGTT | RNAi |  |
| siGFP-2 | AACAAGAATTGGGACAACTCCCCCTATAGTGAGTCGTATTAGTGATC | RNAi |  |
| siGFP-3 | AAGGAGTTGTCCCAATTCTTGCCCTATAGTGAGTCGTATTAGTGATC | RNAi |  |
| siGFP-4 | GATCACTAATACGACTCACTATAGGGCAAGAATTGGGACAACTCCTT | RNAi |  |
| siWSV001-1 | GATCACTAATACGACTCACTATAGGGAAGGGATGGTGTGGATGGTTCTGTTTT | RNAi |  |
| siWSV001-2 | AAAACAGAACCATCCACACCATCCCTTCCCTATAGTGAGTCGTATTAGTGATC | RNAi |  |
| siWSV001-3 | AAAAGGGATGGTGTGGATGGTTCTGTTCCCTATAGTGAGTCGTATTAGTGATC | RNAi |  |
| siWSV001-4 | GATCACTAATACGACTCACTATAGGGAACAGAACCATCCACACCATCCCTTTT | RNAi |  |
| siWSV091-1 | GATCACTAATACGACTCACTATAGGGCAGGGCCGTGTATGAAGCGTCAATTTT | RNAi |  |
| siWSV091-2 | AAAATTGACGCTTCATACACGGCCCTCCCCTATAGTGAGTCGTATTAGTGATC | RNAi |  |
| siWSV091-3 | AACAGGGCCGTGTATGAAGCGTCAATTCCCTATAGTGAGTCGTATTAGTGATC | RNAi |  |
| siWSV091-4 | GATCACTAATACGACTCACTATAGGGAATTGACGCTTCATACACGGCCCTCTT | RNAi |  |
| siWSV271-1 | GATCACTAATACGACTCACTATAGGGGCTCAACATTTAGTGGACATGACAATT | RNAi |  |
| siWSV271-2 | AATTGTCATGTCCACTAAATGTTGAGCCCCTATAGTGAGTCGTATTAGTGATC | RNAi |  |
| siWSV271-3 | AAGCTCAACATTTAGTGGACATGACAACCCTATAGTGAGTCGTATTAGTGATC | RNAi |  |
| siWSV271-4 | GATCACTAATACGACTCACTATAGGGTTGTCATGTCCACTAAATGTTGAGCTT | RNAi |  |
| siWSV277-1 | GATCACTAATACGACTCACTATAGGGTCGAAAGGACCGGAGAGCCTCTTAATT | RNAi |  |
| siWSV277-2 | AATTAAGAGGCTCTCCGGTCCTTTCGACCCTATAGTGAGTCGTATTAGTGATC | RNAi |  |
| siWSV277-3 | AATCGAAAGGACCGGAGAGCCTCTTAACCCTATAGTGAGTCGTATTAGTGATC | RNAi |  |
| siWSV277-4 | GATCACTAATACGACTCACTATAGGGTTAAGAGGCTCTCCGGTCCTTTCGATT | RNAi |  |
| siWSV477-1 | GATCACTAATACGACTCACTATAGGGCCAGTTCCAGTTATCAACATCAAATTT | RNAi |  |
| siWSV477-2 | AAATTTGATGTTGATAACTGGAACTGGCCCTATAGTGAGTCGTATTAGTGATC | RNAi |  |
| siWSV477-3 | AACCAGTTCCAGTTATCAACATCAAATCCCTATAGTGAGTCGTATTAGTGATC | RNAi |  |
| siWSV477-4 | GATCACTAATACGACTCACTATAGGGATTTGATGTTGATAACTGGAACTGGTT | RNAi |  |
